# Postpartum Body Weight and Cardiometabolic Biomarker Trajectories in Women With Gestational Diabetes: A Population‐Based Matched Cohort Study

**DOI:** 10.1111/dom.70994

**Published:** 2026-06-22

**Authors:** Ruya Abdulsalam, Susan A. Jebb, Moscho Michalopoulou, Carmen Piernas, Richard Stevens, Nerys M. Astbury

**Affiliations:** ^1^ Nuffield Department of Primary Care Health Sciences University of Oxford Oxford UK; ^2^ National Institutes for Health Research (NIHR) Oxford Biomedical Research Centre Oxford UK; ^3^ Department of Biochemistry and Molecular Biology II, Faculty of Pharmacy; Centre for Biomedical Research Institute of Nutrition and Food Technology; Biosanitary Research Institute Ibs.Granada; University of Granada Granada Spain; ^4^ CIBER Physiopathology of Obesity and Nutrition (CIBEROBN) Instituto de Salud Carlos III Madrid Spain

**Keywords:** cohort study, database research, gestational diabetes, type 2 diabetes

## Abstract

**Aims:**

To compare postpartum trajectories in body weight and cardiometabolic biomarkers between women with and without GDM, and to explore the moderating effect of progression to type 2 diabetes (T2DM).

**Materials and Methods:**

We used electronic primary care healthcare records and linked hospital episode records of women who gave birth between 2001 and 2021. Each woman with GDM was matched to four women without GDM based on age at delivery and delivery date (±3 months). We fitted mixed‐effects multivariate linear models to map biomarker trajectories and extracted coefficients and 95% confidence intervals (CIs) for each year postpartum.

**Results:**

A total of 43 572 women diagnosed with GDM were matched with 174 288 women without GDM in pregnancy. Across the 15‐year follow‐up, women with GDM weighed more (difference of 5.5 kg [95% CI: 5.3 to 5.7]), had higher HbA1c concentrations (4.4 mmol/mol [95% CI: 4.3 to 4.5]), systolic and diastolic blood pressure (2.6 mmHg [95% CI: 2.4 to 2.7] and 1.7 mmHg [1.6 to 1.8] higher, respectively), and low‐density lipoprotein cholesterol (0.08 [95% CI: 0.06 to 0.10]). However, biomarkers increased at a slower rate during the follow‐up period among women with GDM, except for HbA1c (increase of 9.3 mmol/mol [95% CI: 8.8 to 9.7] and 2.3 mmol/mol [95% CI: 1.9 to 2.6] in the GDM and non‐GDM groups, respectively). Future Progression to type 2 diabetes moderated biomarker trajectories.

**Conclusion:**

Differences in biomarker trajectories in the early postpartum present an opportunity for early risk stratification and targeted prevention among women with previous GDM.

## 1 Introduction

Gestational diabetes mellitus (GDM) is defined as glucose intolerance with first onset or recognition during pregnancy, in the absence of any pre‐pregnancy diabetes [1, 2]. GDM is the most common pregnancy complication worldwide. In the UK, GDM has been estimated to affect between 10% and 23.1% of pregnancies [3, 4, 5], although the actual prevalence of GDM is difficult to estimate because it is under‐reported in both primary and secondary care [6]. GDM increases the risk of adverse perinatal outcomes to mother and infant, including shoulder dystocia, cesarean section or instrumental delivery, and macrosomia [7, 8] but early diagnosis and monitoring reduce these risks [9]. Despite this, postpartum risks persist. Women with a GDM history are still almost 10 times more likely to develop type 2 diabetes compared to women without a history of GDM [10], with approximately a third developing type 2 diabetes within 15 years of pregnancy [11].

GDM is also associated with a significant increase in the risk of cardiovascular disease. A systematic review found that women with a history of GDM in pregnancy were almost twice as likely to develop cardiovascular disease than those who do not develop GDM during pregnancy [12]. This risk was only modestly attenuated when restricting the analysis to women who did not develop subsequent type 2 diabetes [13]. It remains unclear whether the increase in the risk of CVD following GDM is mediated by progression to type 2 diabetes [14].

Type 2 diabetes, cardiovascular disease and GDM share many risk factors, including obesity, hypertension, and hyperlipidaemia [15]. However, it is not fully understood how risk factors accumulate in the postpartum period preceding cardiometabolic dysfunction. Some studies have attempted to map the changes in cardiometabolic biomarkers after GDM, but they have been limited by small sample sizes and/or short follow‐up periods [15, 16, 17, 18]. Understanding the temporal patterns in cardiometabolic disease risk may help identify the clinical phenotypes that could be targeted for preventative interventions. The aim of this study was to characterise patterns of cardiometabolic biomarkers in the postpartum period and compare these patterns between women who developed GDM during pregnancy with those who do not, and to test the moderating effect of type 2 diabetes on the trajectories.

## 2 Materials and Methods

### 2.1 Design and Data Source

This was a matched retrospective cohort study using anonymised patient data from English general practices obtained from the QResearch database (VERSION 47). The database includes demographic information, medical diagnoses, prescriptions, referral and laboratory results from the primary care records, which are linked with NHS Hospital Episode Statistics (HES) and death certificates from the Office for National Statistics (ONS). The protocol was preregistered and is available in the open science framework (OSF) [19].

### 2.2 Ethical Approval and Consent

The QResearch database has ethical approval from the East Midlands Derby Research Ethics Committee (project code 18/EM/0400), which waived the need for written informed consent for collection of de‐identified patient data. In accordance with this ethical approval, the protocol for this study was reviewed and approved by the QResearch Scientific Advisory Committee as part of the Exploring the Long‐term (clinical) Outcomes following a PrEgnancy affected by Gestational Diabetes Mellitus (ELOPE‐GDM) project (reference Q149).

### 2.3 Study Population

Data were obtained for women aged 13–49 years old, who had at least one singleton delivery recorded in the HES, within the study period (1 January 2000–31 December 2021) and were registered at a general practice (GP) that contributed to the QResearch database during that time and had data available for at least one year before the recorded delivery. Patients were excluded if they had any diabetes diagnosis recorded prior to the delivery. The eligible cohort were followed from the delivery date until transfer to another practice that did not contribute to QResearch, death, or the end of the study period (31 December 2021).

### 2.4 Exposure

Exposure was a diagnosis of GDM, defined as the presence of Read code for GDM in the primary care record in the 40 weeks preceding the delivery (Table S1), or a diagnosis of GDM in the HES delivery record, determined by the presence of ICD‐10 codes O244 or O249 in the linked HES record for the delivery episode. Because there are inconsistencies in testing and recording of GDM diagnoses, and to ensure consistency we used the delivery date as the index date (date of study entry) in all of our analyses. In the United Kingdom, the National Institute for Health and Clinical Excellence (NICE) published its first clinical guidelines for the diagnosis of GDM (CG63) in 2008, in which GDM was diagnosed if a patient had fasting plasma glucose of ≥ 7.0 mmol/L or a 2‐h plasma glucose level of ≥ 7.8 mmol/L in a 75 g oral glucose tolerance test [20]. The criteria were updated in 2015 (NICE guideline NG3), and a GDM diagnosis can now be made if a patient has a fasting plasma glucose level of 5.6 mmol/L or a 2‐h plasma glucose level of 7.8 mmol/L [1]. In this study, we defined GDM as any recorded GDM in the healthcare record.

### 2.5 Comparator

Each of the records included in the exposure group was matched with four records of eligible women who had no recorded GDM matched on maternal age at delivery and delivery date (± 3 months).

### 2.6 Outcomes

Our outcomes of interest were measures of cardiometabolic biomarkers collected at any time after the index date. These biomarkers included weight (kg), HbA1c (mmol/mol), total, low‐density lipoprotein (LDL) and high‐density lipoprotein (HDL) cholesterol concentrations (mmol/L) as well as systolic (SBP) and diastolic blood pressure (DBP) (mmHg). We extracted all recorded measurements available for the eligible analytic sample.

### 2.7 Covariates

Using directed acyclic graphs (DAG) (Figure S1) we identified variables which were confounders, mediators and colliders in the causal pathway between GDM and biomarkers of cardiometabolic risk. Only variables identified as true confounders were included as covariates in our models. These included maternal age (years) at delivery grouped into < 20 years, 20–29 years, 30–39 years, ≥ 40 years), pre‐pregnancy body mass index (BMI) (< 18.5 kg/m^2^, 18.5–19.9 kg/m^2^, 20–24.9 kg/m^2^, 25–29.9 kg/m^2^, 30–34.9 kg/m^2^, > 35 kg/m^2^, missing), self‐reported ethnicity from the primary care record (White, Indian, Pakistani, Bangladeshi, Other Asian, Caribbean, Black African, Chinese, other, missing), family history of type 2 diabetes, most recently recorded smoking history (non‐smoker, ex‐smoker, light smoker (1–9 per day), moderate smoker (10–19 per day), heavy smoker (≥ 20 per day, missing), and socioeconomic status (SES) ascertained by Townsend score fifth; split by quintile (with 1 being the least deprived, and 5 being most deprived). All missing values for covariate data were coded as a separate missing category, except for socioeconomic status, where missing values were imputed using the median score for other patients from the same practice.

### 2.8 Statistical Analysis

We calculated descriptive statistics to summarise patient demographics and mean pre‐pregnancy cardiometabolic biomarkers taken in the 12‐month period prior to the index pregnancy. These were reported as means (SD) for normally distributed variables, as median (interquartile range [IQR]) for variables not normally distributed, and as proportions for categorical variables. Where patients had multiple recorded observations for an outcome in one year, we calculated the mean outcome value for that year, such that for each outcome, each patient had one outcome measure per year. A summary of data cleaning is provided in Box S1.

We fitted multivariable mixed‐effect linear regression models to include fixed effects for time, scaled to one year, exposure (GDM), time by exposure interaction and a random effect for participant. We adjusted all analyses for maternal age, ethnicity, socioeconomic status, smoking history, family history of diabetes and pre‐pregnancy BMI. Missing data for covariates were included as a separate category. Biomarker data recorded after 15 years of follow‐up was not included in the regression models, because the recorded data beyond that point was sparse.

From these models, we extracted the estimated regression coefficients and corresponding 95% confidence intervals by GDM status for each postpartum year and presented them graphically as absolute values and as change from the first postpartum year.

Because the people who go on to develop type 2 diabetes in the future may have different risk profiles, and therefore may plausibly have distinctive biomarker trajectories, we explored the moderating effect of a future type 2 diabetes diagnosis by fitting analogous models with additional multiplicative terms and plotted extracted values from these models as described above. We assessed whether our models met linear model assumptions using QQ plots, observed versus predicted value plots, residuals versus predicted value plots, and histograms of residuals.

All statistical analyses were conducted in Stata 18 with *p* ≤ 0.05 used to indicate statistical significance. Model coefficients were plotted in R Studio, using the ggplot2 package. All codes are available on GitHub: https://github.com/nerysastbury/GDM_Trajectories.git.

## 3 Results

We identified records of 43 572 eligible women who delivered at least one singleton between 1 January 2000 and 31 December 2021 and were diagnosed with GDM during pregnancy. These women were individually matched on delivery date and maternal age (±3 months) with 174 288 women who had no current or previous history of GDM, who made up the control group resulting in a total analytical sample of 217 860 (Figure S2).

The GDM group had higher pre‐pregnancy BMI, were more likely to report a family history of diabetes, a greater proportion were from the most deprived regions, and non‐White ethnic backgrounds compared with the comparator group (Table 1).

**TABLE 1:** Characteristics of included patients.

	GDM *N* = 43 572	Non‐GDM *N* = 174 288	All *N* = 217 860
Part 1—Patient demographics			
Age (years), mean (SD)	32.8 (5.2)	32.8 (5.2)	32.8 (5.2)
Age group, *N* (%)			
< 20 years	218 (1)	872 (1)	872 (0)
20–29 years	11 512 (26)	46 048 (26)	46 048 (21)
30–39 years	27 476 (63)	109 904 (63)	109 904 (50)
≥ 40 years	4366 (10)	17 464 (10)	17 464 (8)
BMI (kg/m^2^)^b^	29.8 (6.8)	26.0 (5.8)	26.8 (6.2)
BMI group, *N* (%)			
< 18.5 kg/m^2^	182 (0)	1470 (1)	1652 (1)
18.5–19.9 kg/m^2^	330 (1)	3222 (2)	3552 (2)
20–24.9 kg/m^2^	2777 (6)	19 070 (11)	21 847 (10)
25–29.9 kg/m^2^	3698 (9)	12 301 (7)	15 999 (7)
30–34.9 kg/m^2^	2974 (7)	5717 (3)	8691 (4)
≥ 35 kg/m^2^	2659 (6)	3703 (2)	6362 (3)
Missing	30 952 (71)	128 805 (74)	159 757 (73)
Self‐reported ethnicity, *N* (%)^a^			
White	19 781 (45)	109 705 (63)	109 365 (50)
Indian	2852 (7)	5812 (3)	5782 (3)
Pakistani	3570 (8)	5883 (3)	5816 (3)
Bangladeshi	3986 (9)	3538 (2)	3473 (2)
Other Asian	2078 (5)	3874 (2)	3842 (2)
Caribbean	478 (1)	1796 (1)	1777 (1)
Black African	2119 (5)	6032 (4)	5952 (3)
Chinese	451 (1)	1315 (1)	1313 (1)
Other	2189 (5)	7776 (5)	7745 (4)
Missing	6068 (14)	28 557 (16)	28 486 (13)
Townsend fifth, *N* (%)^c^			
1 (least deprived)	6286 (14.4)	36 539 (21.0)	42 825 (19.7)
2	7630 (17.5)	37 439 (21.5)	45 069 (20.7)
3	9141 (21.0)	35 665 (20.5)	44 806 (20.6)
4	9811 (22.5)	33 491 (19.2)	43 302 (19.9)
5 (most deprived)	10 704 (24.6)	31 154 (17.9)	41 858 (19.2)
Previous pregnancies, *N* (%)^d^			
0	10 212 (23)	45 917 (26)	56 129 (26)
1	10 490 (24)	43 718 (25)	54 208 (25)
2	6520 (15)	23 140 (13)	29 660 (14)
3	3691 (9)	11 053 (6)	14 744 (7)
4	2052 (5)	5308 (3)	7360 (3)
5 or more	2014 (5)	5478 (3)	7492 (3)
Missing	8593 (20)	39 674 (23)	48 267 (22)
Family history of diabetes, *N* (%)			
No	29 637 (68)	142 436 (82)	172 073 (79)
Yes	13 935 (32)	31 852 (18)	45 787 (21)
Smoking status, *N* (%)			
Non‐smoker	30 226 (69)	111 800 (64)	142 026 (65)
Ex‐smoker	5513 (13)	26 718 (15)	32 231 (15)
Light smoker	5565 (13)	26 668 (15)	32 233 (15)
Moderate smoker	281 (1)	1316 (1)	1597 (1)
Heavy smoker	55 (0)	237 (0)	292 (0)
Missing	1932 (4)	7549 (4)	9481 (4)
Part 2—Pre‐pregnancy clinical biomarkers			
Weight (*N*)^e^	14 280	51 780	66 060
Weight (kg)	78.1 (20.0)	69.9 (16.6)	71.7 (17.7)
HbA1c (*N*)	5704	14 111	19 815
HbA1c (mmol/mol)	36.8 (4.4)	34.0 (3.5)	34.8 (4.0)
Total Cholesterol (*N*)	4377	10 291	14 668
Total Cholesterol (mmol/L)	4.8 (0.9)	4.7 (0.9)	4.7 (0.9)
HDL cholesterol (*N*)	4018	9421	13 439
HDL cholesterol (mmol/L)	1.3 (0.3)	1.5 (0.4)	1.4 (0.4)
LDL cholesterol (*N*)	3089	7093	10 182
LDL cholesterol (mmol/L)	2.9 (0.8)	2.7 (0.8)	2.7 (0.8)
Systolic blood pressure (*N*)	19 655	74 866	94 521
Systolic blood pressure (mmHg)	118.8 (12.6)	116.6 (11.9)	117.1 (12.1)
Diastolic blood pressure (*N*)	19 651	74 871	94 522
Diastolic blood pressure (mmHg)	75.6 (9.1)	73.3 (8.8)	73.8 (9.0)

^a^ Self‐reported ethnicities. Percentages are rounded to the nearest whole number.

^b^ Calculated as mean of all available measures recorded in the primary care record for each participant in the year prior to index pregnancy.

^c^ Townsend index fifth, is a measure of social deprivation for a geographical area [21]. A greater Townsend index score indicates a greater degree of deprivation.

^d^ Number of previous pregnancies that resulted in a registrable birth (live or still born) in the HES delivery record.

^e^ Number of participants for whom pre‐pregnancy data was available.

The GDM group also had higher pre‐pregnancy HbA1c, blood pressure and lipid concentrations compared with the non‐GDM group (Table 1). Follow‐up ranged from 0 to 20 years (median 2.9 years, IQR (1.1 to 6.1)).

The number of records used for each biomarker in the models are shown in Table S2, and the group differences are presented in Tables S3–S6. Because the number of patients contributing data beyond 15 years postpartum was small, we censored our analysis at this point.

### 3.1 Body Weight

**FIGURE 1:**
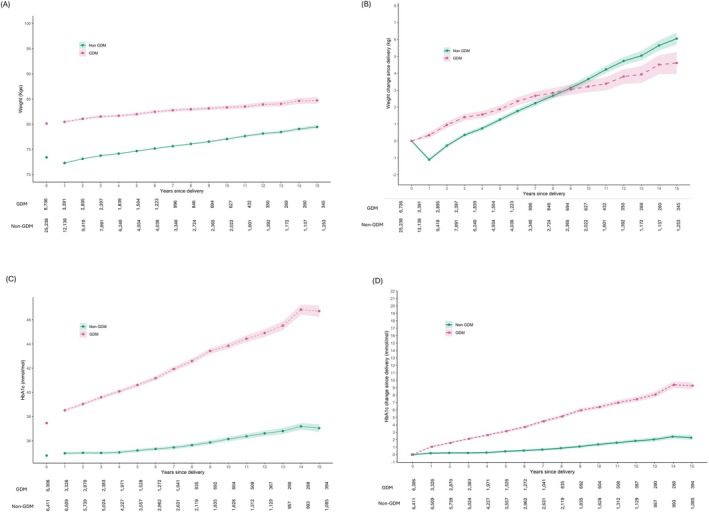
Absolute weight (A) and HbA1c (C) and change from first year postpartum for weight (B) and HbA1c (D) trajectories by GDM status. Plotted biomarker values at each year represent the extracted linear model margins.

Women with GDM were heavier in the first year postpartum (mean difference of 5.0 kg [95% CI: 4.7 to 5.2]) (Figure 1A). Both the GDM and non‐GDM groups gained weight throughout the postpartum period, although the GDM group gained less than the non‐GDM group over the 15‐year follow‐up (weight gain of 4.6 kg [95% CI: 4.0 to 5.3] in the GDM group compared to 6.1 kg [95% CI: 5.7 to 6.4] in the non‐GDM group) (Figure 1B). However, because the GDM group started heavier, they remained heavier than the non‐GDM group throughout the postpartum period (mean difference 5.5 kg [95% CI: 5.3 to 5.7]).

**FIGURE 2:**
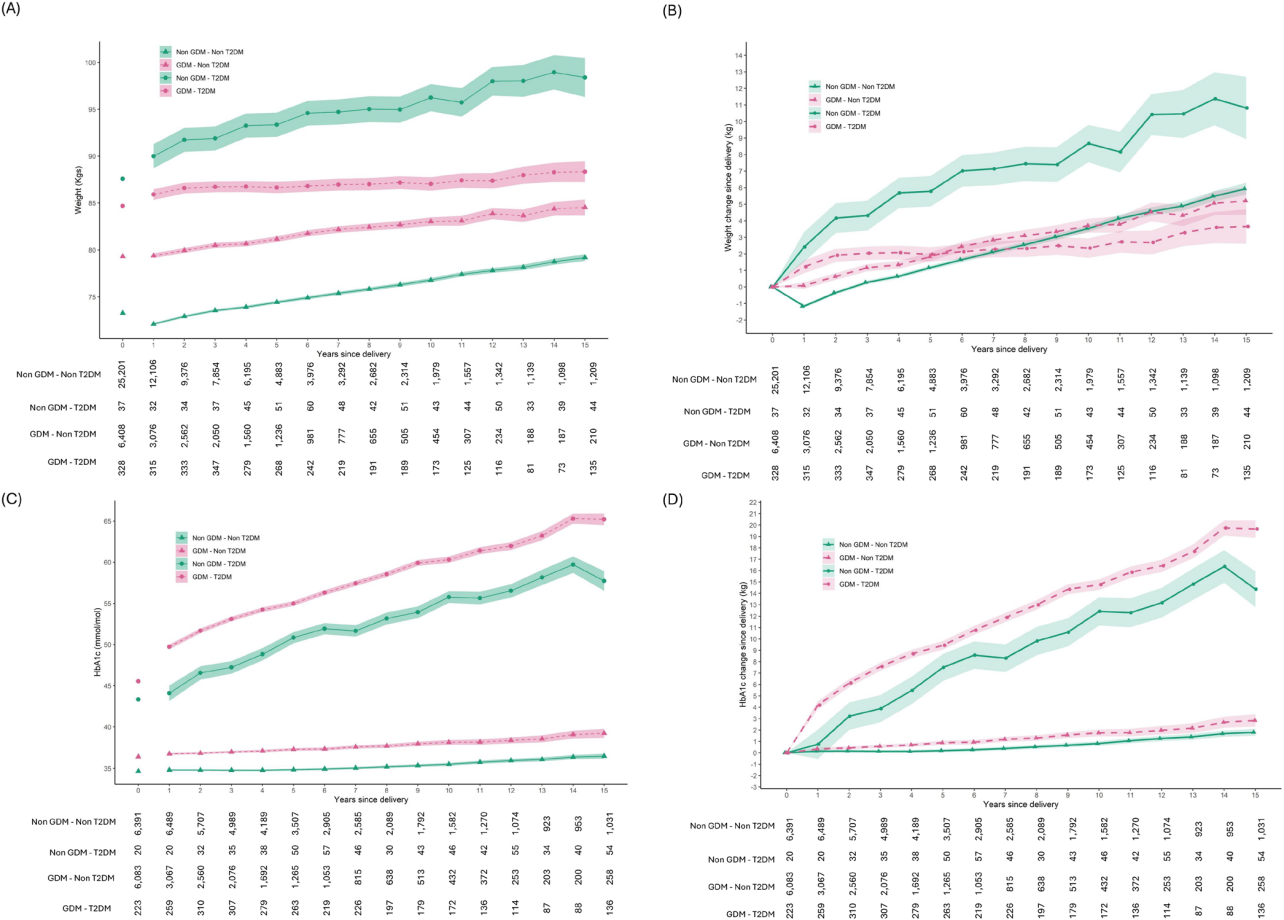
Patterns of absolute weight (A) and HbA1c (C) and change from the first year postpartum in weight (B) and HbA1c (D) by GDM status and progression to type 2 diabetes. Plotted biomarker values at each year represent the extracted linear model margins.

Women without GDM who developed type 2 diabetes had higher average weights across the 15‐year follow‐up compared to women in the GDM group who developed type 2 diabetes. Similarly, weight gain was lower in the GDM group with type 2 diabetes (3.7 kg [95% CI: 2.6 to 4.7]) compared to the non‐GDM group with type 2 diabetes (10.8 kg [95% CI: 8.9 to 12.7]) (Figure 2B).

### 3.2 HbA1c

HbA1c concentrations were higher in the GDM group compared with the non‐GDM group in the first year postpartum (mean difference of 2.1 mmol/mol [95% CI: 1.9 to 2.2]) (Figure 1C). HbA1c concentrations increased in both groups in the postpartum follow‐up period, although there was a greater and more rapid increase in HbA1c among the women with GDM compared with the non‐GDM women (mean increase: 9.3 mmol/mol [95% CI: 8.8 to 9.7 mmol/mol] and 2.3 mmol/mol [95% CI: 1.9 to 2.6 mmol/mol] in the GDM and non‐GDM groups, respectively) (Figure 1D).

The postpartum patterns of absolute HbA1c concentrations in people who did not go on to develop type 2 diabetes were similar regardless of GDM status (Figure 2C). However, there was a clear difference in patterns of HbA1c between those with a history of GDM and non‐GDM in the people who did go on to develop type 2 diabetes (Figure 2D). While HbA1c concentrations increased in both GDM and non‐GDM groups that went on to develop type 2 diabetes, there was a greater increase in HbA1c in those with a history of GDM compared with the non‐GDM group at 15 years after delivery (increase of 19.7 mmol/mol [95% CI: 18.9 to 20.4] and 14.4 mmol/mol [95% CI: 12.8 to 15.9] for women in the GDM and non‐GDM groups who developed type 2 diabetes, respectively).

### 3.3 Blood Lipids

**FIGURE 3:**
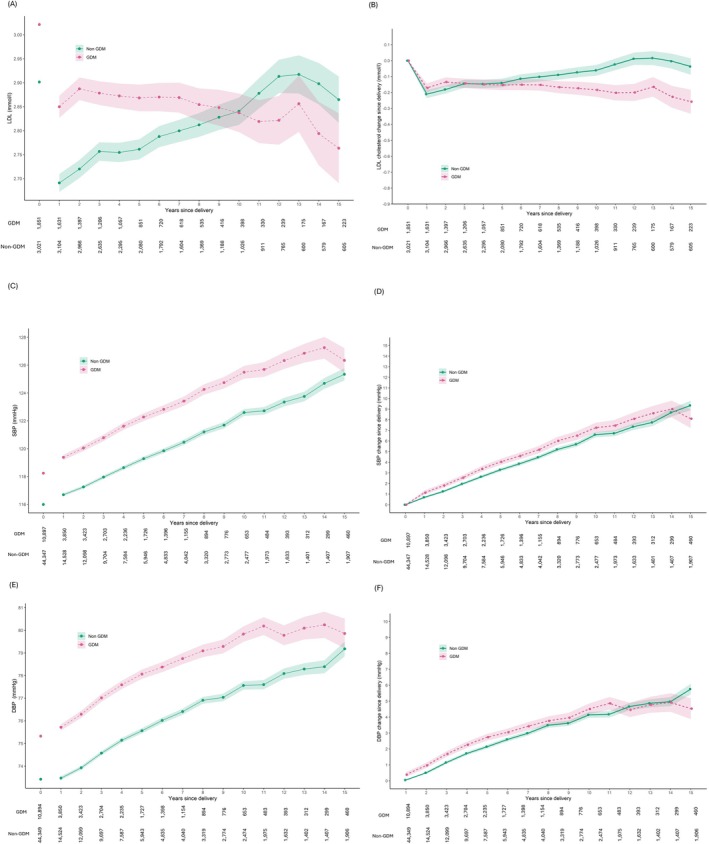
Absolute LDL cholesterol (A), SBP (C) and DBP (E), and change from first year postpartum LDL cholesterol (B), SBP (D) and DBP (F) trajectories by GDM status. Plotted biomarker values at each year represent the extracted linear model margins.

**FIGURE 4:**
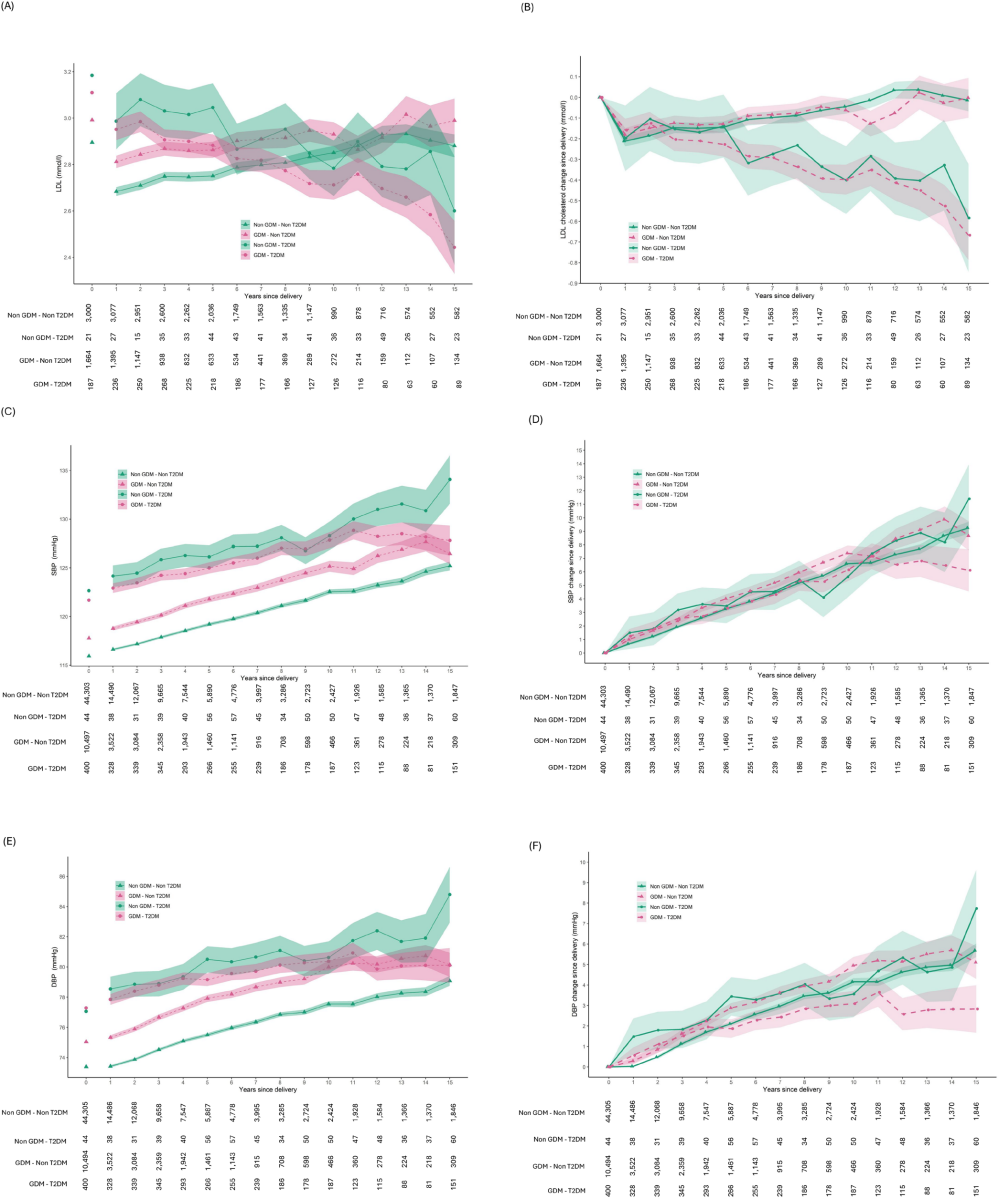
Patterns of absolute LDL cholesterol (A) and SBP (C), DBP (E), and change LDL cholesterol (B), SBP (D) and DBP (F) from first year postpartum by GDM status and type 2 diabetes status. Plotted biomarker values at each year represent the extracted linear model margins.

LDL cholesterol concentrations were higher among the GDM group compared to the non‐GDM group at the first year postpartum (mean difference of 0.11 mmol/L [95% CI 0.08 to 0.14]), and were higher overall over the 15‐year follow‐up (mean difference across 15 years of 0.08 mmol/L [95% CI 0.06 to 0.10]) (Figure 3A). Concentrations of LDL cholesterol decreased more in the GDM group over the 15 years of follow‐up compared to those in the non‐GDM group (difference at 15 years of −0.04 mmol/L [95% CI: −0.09 to 0.01] and −0.26 [95% CI: −0.33 to −0.18] for the non‐GDM and the GDM group, respectively) (Figure 3B). There were no discernible moderating effects of type 2 diabetes on LDL cholesterol concentrations (Figure 4A,B).

Total: HDL ratios, HDL and total cholesterol concentrations followed similar trends and are shown in Figures S3 and S4.

### 3.4 Blood Pressure

SBP and DBP, respectively, displayed similar postpartum patterns, with values higher in the GDM group than in the non‐GDM group in the first year postpartum (mean difference of 2.2 mmHg [95% CI: 2.0 to 2.3] for SBP and 1.4 mmHg [95% CI: 1.3 to 1.6] for DBP) (Figure 3C,E). Blood pressure increased throughout the postpartum period in both GDM and non‐GDM groups. The magnitude of increase was similar between the GDM and non‐GDM groups for both SBP (increase at 15 years by 8.1 mmHg [95% CI: 7.2 to 9.0] for the GDM group and 9.4 mmHg [95% CI: 8.9 to 9.8] in the non‐GDM group) and DBP (increase by 4.5 mmHg [95% CI: 3.9 to 5.2] for the GDM group and 5.8 mmHg [95% CI: 5.4 to 6.1] for the non‐GDM group) (Figure 3D,F). However, because of the differences in blood pressure between the GDM and non‐GDM groups that were evident pre‐pregnancy (Table 1) and in the first year postpartum, absolute blood pressure readings were higher across the 15‐year follow‐up for the GDM group compared with the non‐GDM women (mean difference of 2.6 mmHg [95% CI: 2.4 to 2.7] for SBP and 1.7 mmHg [95% CI: 1.6 to 1.8] for DBP). There was no apparent moderating effect of future progression to type 2 diabetes on postpartum patterns of blood pressure (Figure 4C–F).

## 4 Discussion

In this retrospective cohort study, we found that women with GDM had distinct risk phenotypes which were detectable early in the postpartum period. Examining absolute biomarker trajectories and trajectories of biomarker change since delivery showed that women with GDM who develop type 2 diabetes start the postpartum period with higher weight, LDL cholesterol and blood pressure, but these biomarkers remain relatively stable in this group throughout follow‐up, compared to other groups who experience a much greater increase in cardiometabolic risk biomarkers. Women with previous GDM who develop type 2 diabetes do not necessarily experience significant worsening of cardiometabolic biomarkers postpartum (except for HbA1c, where there is a large increase despite relatively smaller weight gain), but they are at consistently higher risk of cardiometabolic disease from the start of the postpartum period.

HbA1c was the only biomarker for which women with previous GDM who developed type 2 diabetes had both the highest absolute concentrations and the largest increase in concentrations. HbA1c change trajectories follow the same pattern for weight change in women without previous GDM who develop type 2 diabetes, representing the established relationship between weight gain and worsening glycaemia [22, 23]. However, there is a stark difference between weight and HbA1c change trajectories among women with GDM who develop type 2 diabetes. While weight remains relatively stable in this population, their HbA1c concentrations increase faster than those without GDM who develop type 2 diabetes. This worsening glycaemia among women with previous GDM appears less dependent on weight gain than in women without GDM. This finding may explain why clinical trials with interventions that target weight loss often fail to achieve improvements in glycaemic outcomes among women with previous GDM [24], highlighting the need for a novel, precision‐medicine approach to reducing future cardiometabolic risk among women with GDM.

Our results suggest that tracking weight, lipids and blood pressure change over time may not provide an accurate insight into risk of future cardiometabolic disease in this group of women. However, assessing the rate of change of HbA1c may be a good indicator of future risk, emphasising the importance of regular postpartum blood glucose monitoring for women with a history of GDM.

In a previous prospective cohort study in a general population of adults who were healthy at study entry, the trajectories of fasting and 2‐h glucose tolerance test in patients who developed type 2 diabetes and those who did not were compared [25]. Similar to our study, they noted a steady small increase in glycaemia in patients who did not develop diabetes contrasted by a more rapid increase in glycaemia in patients who did develop type 2 diabetes. However, in the general population, glycaemia worsened modestly throughout follow‐up for those who developed type 2 diabetes, but saw a sharp increase in the 2 years prior to diagnosis. In our study, however, glycaemia increased at a faster rate but more steadily, without a sharp increase at a certain point in follow‐up. This may indicate a different pathophysiology to type 2 diabetes risk after pregnancy compared to risk in a non‐pregnant population of men and women, although this hypothesis requires further investigation.

Our findings that systolic and diastolic blood pressure follow a similar trend in both groups, but are consistently higher in women with GDM are contrasted by findings from the Coronary Artery Risk Development in Young Adults (CARDIA) study, which found no differences in cardiometabolic risk factors between women with and without GDM [26]. The CARDIA study also found that total and LDL cholesterol began at higher concentrations in women with GDM, but over time, women in the comparator group surpassed the GDM group with higher concentrations of both biomarkers. This may be due to a more intensive course of postpartum screening and treatment for women in the GDM group. This is especially important as the three‐way interaction analysis in this study found that women who developed type 2 diabetes had the highest total: HDL cholesterol ratios. Future studies should attempt to understand the interaction between postpartum dyslipidaemia and its treatment, and its subsequent association with type 2 diabetes in women with a history of GDM. Contrary to the expectation that with worsening glycaemia comes a decline in HDL cholesterol concentrations [27], women both with and without GDM in this study saw an increase in HDL levels, indicating that while dyslipidaemia may be well managed in women after GDM, dysglycaemia may not be equally well managed.

The study highlights that a patient's cardiometabolic profile early in the postpartum period has the potential to accurately predict future type 2 diabetes risk. Rather than relying on worsening over time, new prediction models may accurately predict type 2 diabetes risk for a patient using early postpartum biomarkers, thereby enabling earlier interventions to prevent cardiometabolic biomarker worsening to begin with. Additionally, this work highlights that despite risk communication of type 2 diabetes risk after GDM to patients, any actions taken to mitigate this risk are insufficient in producing clinically meaningful risk reduction at a population level. This may be because personalised risk communication does not produce meaningful behaviour change, as shown by a systematic review of nine systematic reviews on the topic [28].

There are several strengths to this study. First, the study benefits from a large sample size of 217 860 patients, and a long‐term analysis of biomarkers until 15 years postpartum. This is especially strengthened by the use of mixed effects models in our analysis. These models have been shown to deal with data missingness and poorly balanced datasets under a missing‐at‐random assumption [29]. The use of a random intercept for each patient accounts for patient‐level baseline heterogeneity. Second, our model adjusted for shared risk factors of GDM and type 2 diabetes, which allows us to see the difference in trajectories of these biomarkers independently of those risk factors.

The study also has several limitations. First, we were not able to adjust for family history of cardiovascular disease or hypercholesterolemia, as these were not available through the data which this study used. These are especially important factors for the models we used to map the trajectories of blood pressure and cholesterol.

Second, by nature of using electronic health records, missing data presented a challenge in this study. Unlike traditional observational studies, measurement of the outcome biomarkers likely depended on factors including healthcare utilisation by the patient, clinician decision making, threshold for investigation, and health indications for investigation, and is not missing at random. However, in the case of women with GDM, routine screening and follow‐up is indicated clinically and may lead to a more systematic collection of data. Through our patient and public involvement (PPI) interviews, we identified that women who do not return for GP appointments or tests are likely to be those who without support at home to look after the new‐born, overweight or obese patients, people from low SES, patients with postpartum depression, and those facing culture‐based stigma around diabetes. However, these differences apply to all groups in this study, and since our study compares trajectories between the groups, the differences between the groups remain meaningful.

Third, our analysis used all available observations per patient per year, as opposed to complete‐case analysis. This meant that patients in the study did not necessarily have 15 years of follow‐up. Indeed, the median follow‐up per patient was 2.9 years, IQR (1.1 to 6.1). This may mean that the patient population representing each year may vary from one year to another. However, this limitation is in part addressed by the use of mixed‐effects models as aforementioned.

Fourth, the results of this study may be subject to misclassification bias, as women were grouped based on GDM and type 2 diabetes status, where poor coding of the disease status may contribute to misclassification. In addition, women in the non‐type 2 diabetes group may have gone on to have a subsequent type 2 diabetes diagnosis after the end of the study date. GDM diagnoses in particular have been shown to be poorly coded in primary and secondary care datasets [6]. GDM diagnosis criteria were not formalised in the United Kingdom until 2008, and were subsequently further updated in 2015. The new criteria used lower thresholds for fasting and 2‐h plasma glucose levels. The variation in GDM diagnostic criteria may have introduced further variation to the classification of GDM in this study. Nonetheless, these discrepancies should not impact on the comparisons between groups.

The small number of women with GDM followed up postpartum reflects previously published literature on diabetes screening and prevention programmes for women with previous GDM. The National Gestational Diabetes Audit 2024–2025 found that only 57.4% of women with GDM had an annual HbA1c test, 44.7% had BMI measured, and only 4.5% of them participated in the NHS Diabetes Prevention Programme [6]. Together with the findings of this study, this highlights the need to develop effective interventions to increase uptake of screening and diabetes prevention programmes among women with GDM.

In the future, analyses of events within the patient groups presented in this study would be of interest including type 1 diabetes, dyslipidaemia, hypertension and obesity, and may shed light on how the biomarker trajectories correspond to clinical outcomes in these patients.

This study shows that women with GDM have distinct cardiometabolic risk trajectories that appear to be independent of changes in body weight change. This increased risk is evident since the beginning of the postpartum period, highlighting the opportunity for early risk stratification and preventative interventions.

## Author Contributions

The study was conceived by R.A., M.M., N.M.A. and S.A.J. R.A. conducted the analysis and drafted the initial version of the manuscript in partial fulfilment of a DPhil degree. C.P. reviewed and advised the analysis. R.S. advised on the statistical analyses. All coauthors contributed to review and finalisation of the manuscript.

## Funding

This work was funded by a PhD studentship by Diabetes UK [grant number 22/0006478] and supported by NIHR Applied Research Collaboration Thames Valley. C.P. is funded by RYC2020‐028818‐I (MCIN/AEI/10.13039/501100011033) and ‘ESF Investing in your future’, Ministry of Science and Innovation, Spain and NIHR ARC Oxford & Thames Valley (NIHR209636).

## Conflicts of Interest

The authors declare no conflicts of interest.

## Supporting information


**Data S1:** Supporting Information.

**Table S1:** READ codes used to identify GDM from the primary care record.
**Box S1:** Summary of data cleaning.
**Figure S1:** Direct acyclic diagram used to identify moderators, mediators and colliders for covariate inclusion in the biomarker.
**Figure S2:** Flowchart of patients eligible for inclusion in the study.
**Table S2:** Number of records available at each year of follow‐up per biomarker.
**Table S3:** Differences between GDM and non‐GDM group at 0, 5, 10 and 15 years after delivery using two‐way interaction between years since delivery and GDM, and overall 15‐year average difference between groups.
**Table S4:** Differences between GDM and non‐GDM group and T2DM and no T2DM group at 0, 5, 10 and 15 years after delivery using three‐way interaction between years since delivery, type 2 diabetes and GDM, and overall 15‐year average difference between groups.
**Table S5:** change from baseline at each follow‐up year for women in the GDM group compared to those in the non‐GDM group.
**Table S6:** change from baseline at each follow‐up year for women based on both GDM and T2DM status.
**Figure S3:** Absolute and difference from first year postpartum for total cholesterol, HDL cholesterol, and total: HDL cholesterol ratio among women with or without GDM in pregnancy.
**Figure S4:** Absolute and difference from first year postpartum for total cholesterol, HDL cholesterol, and total: HDL cholesterol ratios among women with or without GDM in pregnancy who did or did not develop type 2 diabetes in pregnancy.

## Data Availability

De‐identified individual participant data that underlie the results reported in this article will be made available, with requests accepted immediately following publication, for proposals that set out to achieve aims specified in a methodologically and scientifically sound protocol that are approved by the QResearch Scientific Advisory Committee (‘learned intermediary’), where costs of providing access to the data are covered, where requests are compliant with the legal permissions of QResearch data providers, and QResearch data security requirements are met. Information regarding submission of applications to access data can be found at www.qresearch.org.
