# Supplementary material for: Postpartum Body Weight and Cardiometabolic Biomarker Trajectories in Women With Gestational Diabetes: A Population‐Based Matched Cohort Study
Source: Diabetes Obes Metab. 2026 Jun 22;28(9):8254–65. doi: 10.1111/dom.70994 (PMC13448872; doi:10.1111/dom.70994)
Supplement: Supplementary file 1 [file DOM-28-8254-s001.docx]

**Postpartum body weight and cardiometabolic biomarker trajectories in women with gestational diabetes: a population-based matched cohort study.**

**Supplementary Material**

Contents

[Supplementary Table 1: READ codes used to identify GDM from the primary care record.	2](#_Toc231039662)

[Supplementary Box 1: Summary of data cleaning.	3](#_Toc231039663)

[Supplementary Figure 1: Direct acyclic diagram used to identify moderators, mediators and colliders for covariate inclusion in the biomarker.	4](#_Toc231039664)

[Supplementary Figure 2: Flowchart of patients eligible for inclusion in the study.	5](#_Toc231039665)

[Supplementary Table 2: Number of records available at each year of follow-up per biomarker.	6](#_Toc231039666)

[Supplementary Table 3. Differences between GDM and non-GDM group at 0, 5, 10 and 15 years after delivery using two-way interaction between years since delivery and GDM, and overall 15-year average difference between groups.	7](#_Toc231039667)

[Supplementary Table 4. Differences between GDM and non-GDM group and T2DM and no T2DM group at 0, 5, 10 and 15 years after delivery using three-way interaction between years since delivery, type 2 diabetes and GDM, and overall 15-year average difference between groups.	9](#_Toc231039668)

[Supplementary Table 5: change from baseline at each follow-up year for women in the GDM group compared to those in the non GDM group.	11](#_Toc231039669)

[Supplementary Table 6: change from baseline at each follow-up year for women based on both GDM and T2DM status.	15](#_Toc231039670)

[Supplementary Figure 3: Absolute and difference from first year postpartum for total cholesterol, HDL cholesterol, and total: HDL cholesterol ratio among women with or without GDM in pregnancy.	21](#_Toc231039671)

[Supplementary Figure 4: Absolute and difference from first year postpartum for total cholesterol, HDL cholesterol, and total: HDL cholesterol ratios among women with or without GDM in pregnancy who did or did not develop type 2 diabetes in pregnancy.	22](#_Toc231039672)

## Supplementary Table 1: READ codes used to identify GDM from the primary care record.

**Gestational diabetes mellitus (GDM)**

| **66Ay** | Gestational diabetes mellitus annual review | 1 | - |  |
| --- | --- | --- | --- | --- |
| **L180** | Diabetes mellitus during pregnancy/childbirth/puerperium | 1 | - |  |
| **L1801** | Diabetes mellitus during pregnancy - baby delivered | 1 | - |  |
| **L1803** | Diabetes mellitus during pregnancy - baby not yet delivered | 1 | - |  |
| **L1808-1** | Gestational diabetes mellitus | 1 | - |  |
| **L1809** | Gestational diabetes mellitus |  |  |  |

## Supplementary Box 1: Summary of data cleaning.

To clean the data, we dropped any biomarker observations taken before the delivery, and dropped any values that were outside of physiologically feasible values (<10 kg/m^2^ or >70 kg/m^2^ for BMI, <10 mmol/mol or >100 mmol/mol for HbA1c, <40 kgs or >300 kgs for weight, <2 mmol/l or >15 mmol/l for total cholesterol, <0.5 mmol/l or >5 mmol/l for HDL cholesterol, <0.2 mmol/l or >10 mmol/l for LDL cholesterol, <60 mmHg or >300 mmHg for SBP, and <30 mmHg or >200 mmHg for DBP).

For pre-pregnancy values, we dropped any measurements after conception or >1 year prior to the conception date. If there was more than one measurement available for a patient, we calculated the mean of those measurements.

Missing deprivation data for an individual patient were imputed using the median of the Townsends recorded for the practice in which the patient is registered. For all other covariates, missing data was included as a separate category.

For all biomarkers, we tested unit uniformity and normality of recorded data.

## Supplementary Figure 1: Direct acyclic diagram used to identify moderators, mediators and colliders for covariate inclusion in the biomarker.

## Supplementary Figure 2: Flowchart of patients eligible for inclusion in the study.

## Supplementary Table 2: Number of records available at each year of follow-up per biomarker.

|  | **Weight** | | | | **HbA1c** | | | | **LDL Cholesterol** | | | | **Systolic blood pressure** | | | | **Diastolic blood pressure** | | | |  |
| --- | --- | --- | --- | --- | --- | --- | --- | --- | --- | --- | --- | --- | --- | --- | --- | --- | --- | --- | --- | --- | --- |
| **Year** | Non GDM - Non T2DM | Non GDM - T2DM | GDM - Non T2DM | GDM - T2DM | Non GDM - Non T2DM | Non GDM - T2DM | GDM - Non T2DM | GDM - T2DM | Non GDM - Non T2DM | Non GDM - T2DM | GDM - Non T2DM | GDM - T2DM | Non GDM - Non T2DM | Non GDM - T2DM | GDM - Non T2DM | GDM - T2DM | Non GDM - Non T2DM | Non GDM - T2DM | GDM - Non T2DM | GDM - T2DM | |
| **0** | 25,201 | 37 | 6,408 | 328 | 6,391 | 20 | 6,083 | 223 | 3,000 | 21 | 1,664 | 187 | 44,303 | 44 | 10,497 | 400 | 44,305 | 44 | 10,494 | 400 | |
| **1** | 12,106 | 32 | 3,076 | 315 | 6,489 | 20 | 3,067 | 259 | 3,077 | 27 | 1,395 | 236 | 14,490 | 38 | 3,522 | 328 | 14,486 | 38 | 3,522 | 328 | |
| **2** | 9,376 | 34 | 2,562 | 333 | 5,707 | 32 | 2,560 | 310 | 2,951 | 15 | 1,147 | 250 | 12,067 | 31 | 3,084 | 339 | 12,068 | 31 | 3,084 | 339 | |
| **3** | 7,854 | 37 | 2,050 | 347 | 4,989 | 35 | 2,076 | 307 | 2,600 | 35 | 938 | 268 | 9,665 | 39 | 2,358 | 345 | 9,658 | 39 | 2,359 | 345 | |
| **4** | 6,195 | 45 | 1,560 | 279 | 4,189 | 38 | 1,692 | 279 | 2,262 | 33 | 832 | 225 | 7,544 | 40 | 1,943 | 293 | 7,547 | 40 | 1,942 | 293 | |
| **5** | 4,883 | 51 | 1,236 | 268 | 3,507 | 50 | 1,265 | 263 | 2,036 | 44 | 633 | 218 | 5,890 | 56 | 1,460 | 266 | 5,887 | 56 | 1,461 | 266 | |
| **6** | 3,976 | 60 | 981 | 242 | 2,905 | 57 | 1,053 | 219 | 1,749 | 43 | 534 | 186 | 4,776 | 57 | 1,141 | 255 | 4,778 | 57 | 1,143 | 255 | |
| **7** | 3,292 | 48 | 777 | 219 | 2,585 | 46 | 815 | 226 | 1,563 | 41 | 441 | 177 | 3,997 | 45 | 916 | 239 | 3,995 | 45 | 915 | 239 | |
| **8** | 2,682 | 42 | 655 | 191 | 2,089 | 30 | 638 | 197 | 1,335 | 34 | 369 | 166 | 3,286 | 34 | 708 | 186 | 3,285 | 34 | 708 | 186 | |
| **9** | 2,314 | 51 | 505 | 189 | 1,792 | 43 | 513 | 179 | 1,147 | 41 | 289 | 127 | 2,723 | 50 | 598 | 178 | 2,724 | 50 | 598 | 178 | |
| **10** | 1,979 | 43 | 454 | 173 | 1,582 | 46 | 432 | 172 | 990 | 36 | 272 | 126 | 2,427 | 50 | 466 | 187 | 2,424 | 50 | 466 | 187 | |
| **11** | 1,557 | 44 | 307 | 125 | 1,270 | 42 | 372 | 136 | 878 | 33 | 214 | 116 | 1,926 | 47 | 361 | 123 | 1,928 | 47 | 360 | 123 | |
| **12** | 1,342 | 50 | 234 | 116 | 1,074 | 55 | 253 | 114 | 716 | 49 | 159 | 80 | 1,585 | 48 | 278 | 115 | 1,584 | 48 | 278 | 115 | |
| **13** | 1,139 | 33 | 188 | 81 | 923 | 34 | 203 | 87 | 574 | 26 | 112 | 63 | 1,365 | 36 | 224 | 88 | 1,366 | 36 | 224 | 88 | |
| **14** | 1,098 | 39 | 187 | 73 | 953 | 40 | 200 | 88 | 552 | 27 | 107 | 60 | 1,370 | 37 | 218 | 81 | 1,370 | 37 | 218 | 81 | |
| **15** | 1,209 | 44 | 210 | 135 | 1,031 | 54 | 258 | 136 | 582 | 23 | 134 | 89 | 1,847 | 60 | 309 | 151 | 1,846 | 60 | 309 | 151 | |

## Supplementary Table 3. Differences between GDM and non-GDM group at 0, 5, 10 and 15 years after delivery using two-way interaction between years since delivery and GDM, and overall 15-year average difference between groups.

| Difference | Comparison group | dy/dx | 95% confidence interval | |
| --- | --- | --- | --- | --- |
| Weight (Kg) | | | | |
| Difference at years since delivery = 0 | 1.GDM | 4.95 | 4.71 | 5.20 |
| Difference at years since delivery = 5 | 1.GDM | 5.57 | 5.26 | 5.88 |
| Difference at years since delivery = 10 | 1.GDM | 4.51 | 4.08 | 4.94 |
| Difference at years since delivery = 15 | 1.GDM | 3.51 | 2.75 | 4.26 |
| Average difference across 15 years | 1.GDM | 5.51 | 5.28 | 5.74 |
| HbA1c (mmol/mol) | | | | |
| Difference at years since delivery = 0 | 1.GDM | 2.09 | 1.94 | 2.23 |
| Difference at years since delivery = 5 | 1.GDM | 4.80 | 4.60 | 5.01 |
| Difference at years since delivery = 10 | 1.GDM | 7.10 | 6.80 | 7.41 |
| Difference at years since delivery = 15 | 1.GDM | 9.07 | 8.51 | 9.64 |
| Average difference across 15 years | 1.GDM | 4.39 | 4.29 | 4.48 |
| Total: HDL cholesterol (ratio) | | | | |
| Difference at years since delivery = 0 | 1.GDM | 0.37 | 0.33 | 0.40 |
| Difference at years since delivery = 5 | 1.GDM | 0.37 | 0.33 | 0.40 |
| Difference at years since delivery = 10 | 1.GDM | 0.26 | 0.21 | 0.31 |
| Difference at years since delivery = 15 | 1.GDM | 0.23 | 0.15 | 0.31 |
| Average difference across 15 years | 1.GDM | 0.37 | 0.35 | 0.39 |
| SBP (mmHg) | | | | |
| Difference at years since delivery = 0 | 1.GDM | 2.16 | 2.00 | 2.33 |
| Difference at years since delivery = 5 | 1.GDM | 2.91 | 2.59 | 3.22 |
| Difference at years since delivery = 10 | 1.GDM | 2.81 | 2.29 | 3.33 |
| Difference at years since delivery = 15 | 1.GDM | 0.92 | -0.07 | 1.90 |
| Average difference across 15 years | 1.GDM | 2.58 | 2.44 | 2.72 |
| DBP (mmHg) | | | | |
| Difference at years since delivery = 0 | 1.GDM | 1.44 | 1.32 | 1.56 |
| Difference at years since delivery = 5 | 1.GDM | 2.04 | 1.80 | 2.27 |
| Difference at years since delivery = 10 | 1.GDM | 1.81 | 1.42 | 2.19 |
| Difference at years since delivery = 15 | 1.GDM | 0.21 | -0.52 | 0.94 |
| Average difference across 15 years | 1.GDM | 1.73 | 1.63 | 1.83 |
| LDL cholesterol (mmol/l) | | | | |
| Difference at years since delivery = 0 | 1.GDM | 0.11 | 0.08 | 0.14 |
| Difference at years since delivery = 5 | 1.GDM | 0.10 | 0.06 | 0.13 |
| Difference at years since delivery = 10 | 1.GDM | -0.01 | -0.06 | 0.04 |
| Difference at years since delivery = 15 | 1.GDM | -0.11 | -0.20 | -0.02 |
| Average difference across 15 years | 1.GDM | 0.08 | 0.06 | 0.10 |
| HDL cholesterol (mmol/l) | | | | |
| Difference at years since delivery = 0 | 1.GDM | -0.09 | -0.10 | -0.08 |
| Difference at years since delivery = 5 | 1.GDM | -0.12 | -0.14 | -0.11 |
| Difference at years since delivery = 10 | 1.GDM | -0.13 | -0.15 | -0.11 |
| Difference at years since delivery = 15 | 1.GDM | -0.15 | -0.18 | -0.13 |
| Average difference across 15 years | 1.GDM | -0.12 | -0.13 | -0.11 |
| Total cholesterol (mmol/l) | | | | |
| Difference at years since delivery = 0 | 1.GDM | 0.16 | 0.13 | 0.19 |
| Difference at years since delivery = 5 | 1.GDM | 0.09 | 0.05 | 0.12 |
| Difference at years since delivery = 10 | 1.GDM | -0.04 | -0.08 | 0.01 |
| Difference at years since delivery = 15 | 1.GDM | -0.15 | -0.23 | -0.07 |
| Average difference across 15 years | 1.GDM | 0.09 | 0.07 | 0.11 |

## Supplementary Table 4. Differences between GDM and non-GDM group and T2DM and no T2DM group at 0, 5, 10 and 15 years after delivery using three-way interaction between years since delivery, type 2 diabetes and GDM, and overall 15-year average difference between groups.

| Difference | Comparison | dy/dx | 95% confidence interval | |
| --- | --- | --- | --- | --- |
| Weight (kg) | | | | |
| Difference at years since delivery = 0 | 1.GDM | 4.11 | 3.85 | 4.37 |
|  | 1.T2DM | 9.42 | 8.46 | 10.38 |
| Difference at years since delivery = 5 | 1.GDM | 4.47 | 4.14 | 4.81 |
|  | 1.T2DM | 12.95 | 11.94 | 13.95 |
| Difference at years since delivery = 10 | 1.GDM | 3.85 | 3.37 | 4.32 |
|  | 1.T2DM | 12.98 | 11.85 | 14.11 |
| Difference at years since delivery = 15 | 1.GDM | 2.94 | 2.07 | 3.82 |
|  | 1.T2DM | 12.74 | 11.10 | 14.37 |
| Average difference across 15 years | 1.GDM | 4.42 | 4.18 | 4.66 |
|  | 1.T2DM | 11.99 | 11.12 | 12.85 |
| HbA1c (mmol/mol) | | | | |
| Difference at years since delivery = 0 | 1.GDM | 1.51 | 1.36 | 1.67 |
|  | 1.T2DM | 8.41 | 7.78 | 9.04 |
| Difference at years since delivery = 5 | 1.GDM | 2.32 | 2.13 | 2.52 |
|  | 1.T2DM | 16.22 | 15.79 | 16.65 |
| Difference at years since delivery = 10 | 1.GDM | 2.57 | 2.27 | 2.88 |
|  | 1.T2DM | 20.58 | 20.11 | 21.06 |
| Difference at years since delivery = 15 | 1.GDM | 2.95 | 2.38 | 3.51 |
|  | 1.T2DM | 22.71 | 21.90 | 23.51 |
| Average difference across 15 years | 1.GDM | 2.18 | 2.10 | 2.26 |
|  | 1.T2DM | 14.17 | 13.93 | 14.41 |
| Total cholesterol (mmol/l) | | | | |
| Difference at years since delivery = 0 | 1.GDM | 0.12 | 0.09 | 0.15 |
|  | 1.T2DM | 0.20 | 0.12 | 0.29 |
| Difference at years since delivery = 5 | 1.GDM | 0.06 | 0.03 | 0.10 |
|  | 1.T2DM | 0.16 | 0.09 | 0.23 |
| Difference at years since delivery = 10 | 1.GDM | 0.03 | -0.02 | 0.09 |
|  | 1.T2DM | -0.12 | -0.20 | -0.04 |
| Difference at years since delivery = 15 | 1.GDM | 0.05 | -0.03 | 0.14 |
|  | 1.T2DM | -0.49 | -0.62 | -0.36 |
| Average difference across 15 years | 1.GDM | 0.09 | 0.07 | 0.10 |
|  | 1.T2DM | 0.11 | 0.06 | 0.15 |
| SBP (mmHg) | | | | |
| Difference at years since delivery = 0 | 1.GDM | 1.75 | 1.57 | 1.92 |
|  | 1.T2DM | 5.30 | 4.53 | 6.07 |
| Difference at years since delivery = 5 | 1.GDM | 2.45 | 2.11 | 2.79 |
|  | 1.T2DM | 5.30 | 4.37 | 6.24 |
| Difference at years since delivery = 10 | 1.GDM | 2.51 | 1.92 | 3.09 |
|  | 1.T2DM | 4.26 | 3.09 | 5.42 |
| Difference at years since delivery = 15 | 1.GDM | 0.91 | -0.20 | 2.03 |
|  | 1.T2DM | 6.40 | 4.39 | 8.41 |
| Average difference across 15 years | 1.GDM | 2.10 | 1.96 | 2.25 |
|  | 1.T2DM | 5.56 | 5.00 | 6.12 |
| DBP (mmHg) | | | | |
| Difference at years since delivery = 0 | 1.GDM | 1.24 | 1.12 | 1.37 |
|  | 1.T2DM | 2.50 | 1.94 | 3.06 |
| Difference at years since delivery = 5 | 1.GDM | 1.89 | 1.64 | 2.14 |
|  | 1.T2DM | 3.31 | 2.62 | 4.00 |
| Difference at years since delivery = 10 | 1.GDM | 1.97 | 1.54 | 2.41 |
|  | 1.T2DM | 1.62 | 0.76 | 2.49 |
| Difference at years since delivery = 15 | 1.GDM | 0.43 | -0.40 | 1.25 |
|  | 1.T2DM | 3.58 | 2.09 | 5.07 |
| Average difference across 15 years | 1.GDM | 1.51 | 1.41 | 1.62 |
|  | 1.T2DM | 2.91 | 2.51 | 3.31 |
| LDL cholesterol (mmol/l) | | | | |
| Difference at years since delivery = 0 | 1.GDM | 0.07 | 0.04 | 0.11 |
|  | 1.T2DM | 0.20 | 0.10 | 0.28 |
| Difference at years since delivery = 5 | 1.GDM | 0.07 | 0.04 | 0.11 |
|  | 1.T2DM | 0.16 | 0.09 | 0.23 |
| Difference at years since delivery = 10 | 1.GDM | 0.06 | 0.00 | 0.11 |
|  | 1.T2DM | -0.16 | -0.24 | -0.07 |
| Difference at years since delivery = 15 | 1.GDM | 0.07 | -0.03 | 0.17 |
|  | 1.T2DM | -0.41 | -0.58 | -0.25 |
| Average difference across 15 years | 1.GDM | 0.08 | 0.06 | 0.10 |
|  | 1.T2DM | 0.09 | 0.05 | 0.14 |
| HDL cholesterol (mmol/l) | | | | |
| Difference at years since delivery = 0 | 1.GDM | -0.06 | -0.07 | -0.05 |
|  | 1.T2DM | -0.18 | -0.22 | -0.15 |
| Difference at years since delivery = 5 | 1.GDM | -0.09 | -0.10 | -0.08 |
|  | 1.T2DM | -0.22 | -0.24 | -0.19 |
| Difference at years since delivery = 10 | 1.GDM | -0.09 | -0.10 | -0.07 |
|  | 1.T2DM | -0.23 | -0.26 | -0.20 |
| Difference at years since delivery = 15 | 1.GDM | -0.10 | -0.13 | -0.07 |
|  | 1.T2DM | -0.26 | -0.31 | -0.22 |
| Average difference across 15 years | 1.GDM | -0.08 | -0.09 | -0.08 |
|  | 1.T2DM | -0.21 | -0.23 | -0.20 |
| Total: HDL cholesterol (ratio) | | | | |
| Difference at years since delivery = 0 | 1.GDM | 0.26 | 0.22 | 0.30 |
|  | 1.T2DM | 0.67 | 0.57 | 0.77 |
| Difference at years since delivery = 5 | 1.GDM | 0.25 | 0.21 | 0.28 |
|  | 1.T2DM | 0.76 | 0.68 | 0.83 |
| Difference at years since delivery = 10 | 1.GDM | 0.20 | 0.15 | 0.26 |
|  | 1.T2DM | 0.48 | 0.39 | 0.57 |
| Difference at years since delivery = 15 | 1.GDM | 0.26 | 0.17 | 0.36 |
|  | 1.T2DM | 0.31 | 0.16 | 0.45 |
| Average difference across 15 years | 1.GDM | 0.26 | 0.24 | 0.28 |
|  | 1.T2DM | 0.71 | 0.65 | 0.76 |

## Supplementary Table 5: change from baseline at each follow-up year for women in the GDM group compared to those in the non GDM group.

| **Weight change since delivery** | | | | | | |
| --- | --- | --- | --- | --- | --- | --- |
|  | Non GDM | | | GDM | | |
| Years since delivery | Weight change (Kg) | 95% CI lower bound | 95% CI upper bound | Weight change (Kg) | 95% CI lower bound | 95% CI upper bound |
| 1 | -1.11 | -1.21 | -1.01 | 0.34 | 0.17 | 0.51 |
| 2 | -0.28 | -0.38 | -0.17 | 0.95 | 0.77 | 1.13 |
| 3 | 0.35 | 0.24 | 0.46 | 1.41 | 1.22 | 1.60 |
| 4 | 0.74 | 0.62 | 0.86 | 1.56 | 1.35 | 1.77 |
| 5 | 1.26 | 1.13 | 1.39 | 1.88 | 1.66 | 2.10 |
| 6 | 1.76 | 1.62 | 1.90 | 2.35 | 2.11 | 2.59 |
| 7 | 2.23 | 2.07 | 2.38 | 2.66 | 2.41 | 2.92 |
| 8 | 2.67 | 2.50 | 2.84 | 2.84 | 2.56 | 3.12 |
| 9 | 3.13 | 2.95 | 3.31 | 3.04 | 2.74 | 3.35 |
| 10 | 3.66 | 3.46 | 3.85 | 3.22 | 2.88 | 3.55 |
| 11 | 4.23 | 4.01 | 4.45 | 3.38 | 2.99 | 3.77 |
| 12 | 4.73 | 4.49 | 4.97 | 3.81 | 3.37 | 4.25 |
| 13 | 5.04 | 4.77 | 5.31 | 3.93 | 3.43 | 4.43 |
| 14 | 5.65 | 5.35 | 5.95 | 4.51 | 3.95 | 5.08 |
| 15 | 6.05 | 5.70 | 6.40 | 4.61 | 3.96 | 5.26 |
| **HbA1c change since delivery** | | | | | | |
|  | Non GDM | | | GDM | | |
| Years since delivery | HbA1c change (mmol/mol) | 95% CI lower bound | 95% CI upper bound | HbA1c change (mmol/mol) | 95% CI lower bound | 95% CI upper bound |
| 1 | 0.19 | 0.05 | 0.32 | 1.05 | 0.92 | 1.18 |
| 2 | 0.23 | 0.09 | 0.37 | 1.58 | 1.44 | 1.71 |
| 3 | 0.22 | 0.07 | 0.36 | 2.13 | 1.98 | 2.27 |
| 4 | 0.27 | 0.12 | 0.42 | 2.62 | 2.46 | 2.78 |
| 5 | 0.43 | 0.27 | 0.58 | 3.14 | 2.97 | 3.32 |
| 6 | 0.55 | 0.38 | 0.72 | 3.71 | 3.53 | 3.90 |
| 7 | 0.67 | 0.50 | 0.85 | 4.47 | 4.27 | 4.67 |
| 8 | 0.86 | 0.67 | 1.05 | 5.13 | 4.92 | 5.35 |
| 9 | 1.09 | 0.89 | 1.29 | 5.96 | 5.73 | 6.20 |
| 10 | 1.37 | 1.16 | 1.58 | 6.39 | 6.13 | 6.65 |
| 11 | 1.60 | 1.37 | 1.83 | 6.98 | 6.69 | 7.27 |
| 12 | 1.84 | 1.59 | 2.09 | 7.44 | 7.12 | 7.77 |
| 13 | 2.03 | 1.76 | 2.31 | 8.06 | 7.69 | 8.43 |
| 14 | 2.42 | 2.12 | 2.72 | 9.39 | 8.97 | 9.81 |
| 15 | 2.27 | 1.94 | 2.60 | 9.26 | 8.78 | 9.74 |
| **LDL cholesterol change since delivery** | | | | | | |
|  | Non GDM | | | GDM | | |
| Years since delivery | LDL cholesterol (mmol/l) | 95% CI lower bound | 95% CI upper bound | LDL cholesterol (mmol/l) | 95% CI lower bound | 95% CI upper bound |
| 1 | -0.21 | -0.24 | -0.19 | -0.17 | -0.20 | -0.14 |
| 2 | -0.18 | -0.21 | -0.16 | -0.13 | -0.16 | -0.11 |
| 3 | -0.14 | -0.17 | -0.12 | -0.14 | -0.17 | -0.11 |
| 4 | -0.15 | -0.17 | -0.12 | -0.15 | -0.18 | -0.12 |
| 5 | -0.14 | -0.17 | -0.11 | -0.15 | -0.19 | -0.12 |
| 6 | -0.11 | -0.14 | -0.09 | -0.15 | -0.19 | -0.12 |
| 7 | -0.10 | -0.13 | -0.07 | -0.15 | -0.19 | -0.12 |
| 8 | -0.09 | -0.12 | -0.06 | -0.17 | -0.20 | -0.13 |
| 9 | -0.07 | -0.10 | -0.04 | -0.17 | -0.21 | -0.13 |
| 10 | -0.06 | -0.09 | -0.03 | -0.18 | -0.23 | -0.14 |
| 11 | -0.02 | -0.06 | 0.01 | -0.20 | -0.25 | -0.15 |
| 12 | 0.01 | -0.03 | 0.05 | -0.20 | -0.25 | -0.15 |
| 13 | 0.02 | -0.03 | 0.06 | -0.17 | -0.23 | -0.10 |
| 14 | 0.00 | -0.05 | 0.04 | -0.23 | -0.30 | -0.16 |
| 15 | -0.04 | -0.09 | 0.01 | -0.26 | -0.33 | -0.18 |
| **HDL cholesterol change since delivery** | | | | | | |
|  | Non GDM | | | GDM | | |
| Years since delivery | HDL cholesterol (mmol/l) | 95% CI lower bound | 95% CI upper bound | HDL cholesterol (mmol/l) | 95% CI lower bound | 95% CI upper bound |
| 1 | -0.04 | -0.04 | -0.03 | -0.05 | -0.06 | -0.04 |
| 2 | -0.02 | -0.03 | -0.01 | -0.04 | -0.05 | -0.03 |
| 3 | -0.01 | -0.02 | 0.00 | -0.04 | -0.05 | -0.03 |
| 4 | 0.01 | 0.00 | 0.02 | -0.03 | -0.04 | -0.02 |
| 5 | 0.02 | 0.01 | 0.03 | -0.01 | -0.02 | 0.00 |
| 6 | 0.04 | 0.03 | 0.05 | -0.01 | -0.02 | 0.00 |
| 7 | 0.05 | 0.04 | 0.06 | 0.00 | -0.02 | 0.01 |
| 8 | 0.05 | 0.04 | 0.06 | 0.01 | 0.00 | 0.02 |
| 9 | 0.07 | 0.06 | 0.08 | 0.01 | 0.00 | 0.02 |
| 10 | 0.07 | 0.06 | 0.08 | 0.03 | 0.02 | 0.04 |
| 11 | 0.06 | 0.05 | 0.08 | 0.02 | 0.01 | 0.04 |
| 12 | 0.07 | 0.06 | 0.08 | 0.04 | 0.03 | 0.06 |
| 13 | 0.08 | 0.06 | 0.09 | 0.05 | 0.03 | 0.07 |
| 14 | 0.07 | 0.06 | 0.09 | 0.03 | 0.01 | 0.05 |
| 15 | 0.09 | 0.07 | 0.10 | 0.03 | 0.00 | 0.05 |
| **Total cholesterol change since delivery** | | | | | | |
|  | Non GDM | | | GDM | | |
| Years since delivery | Total cholesterol (mmol/l) | 95% CI lower bound | 95% CI upper bound | Total cholesterol (mmol/l) | 95% CI lower bound | 95% CI upper bound |
| 1 | -0.28 | -0.31 | -0.26 | -0.26 | -0.28 | -0.23 |
| 2 | -0.23 | -0.26 | -0.21 | -0.22 | -0.25 | -0.19 |
| 3 | -0.18 | -0.20 | -0.15 | -0.21 | -0.23 | -0.18 |
| 4 | -0.15 | -0.18 | -0.13 | -0.20 | -0.23 | -0.17 |
| 5 | -0.12 | -0.14 | -0.09 | -0.19 | -0.22 | -0.16 |
| 6 | -0.08 | -0.11 | -0.05 | -0.15 | -0.18 | -0.12 |
| 7 | -0.03 | -0.06 | -0.01 | -0.14 | -0.18 | -0.11 |
| 8 | -0.01 | -0.04 | 0.01 | -0.13 | -0.17 | -0.10 |
| 9 | 0.03 | 0.00 | 0.06 | -0.12 | -0.16 | -0.08 |
| 10 | 0.07 | 0.04 | 0.10 | -0.13 | -0.17 | -0.08 |
| 11 | 0.12 | 0.08 | 0.15 | -0.11 | -0.16 | -0.07 |
| 12 | 0.16 | 0.12 | 0.19 | -0.09 | -0.14 | -0.04 |
| 13 | 0.20 | 0.16 | 0.24 | -0.07 | -0.12 | -0.01 |
| 14 | 0.21 | 0.17 | 0.25 | -0.07 | -0.13 | -0.01 |
| 15 | 0.20 | 0.15 | 0.25 | -0.11 | -0.18 | -0.04 |
| **Total : HDL cholesterol ratio change since delivery** | | | | | | |
|  | Non GDM | | | GDM | | |
| Years since delivery | Ratio change (mmol/l) | 95% CI lower bound | 95% CI upper bound | Ratio change (mmol/l) | 95% CI lower bound | 95% CI upper bound |
| 1 | -0.13 | -0.15 | -0.10 | -0.07 | -0.10 | -0.05 |
| 2 | -0.12 | -0.14 | -0.09 | -0.05 | -0.08 | -0.02 |
| 3 | -0.10 | -0.13 | -0.08 | -0.06 | -0.09 | -0.03 |
| 4 | -0.12 | -0.15 | -0.10 | -0.09 | -0.12 | -0.06 |
| 5 | -0.12 | -0.15 | -0.10 | -0.12 | -0.15 | -0.09 |
| 6 | -0.13 | -0.15 | -0.10 | -0.08 | -0.12 | -0.05 |
| 7 | -0.11 | -0.14 | -0.08 | -0.11 | -0.14 | -0.07 |
| 8 | -0.09 | -0.12 | -0.06 | -0.13 | -0.17 | -0.09 |
| 9 | -0.13 | -0.16 | -0.10 | -0.12 | -0.16 | -0.08 |
| 10 | -0.10 | -0.13 | -0.06 | -0.20 | -0.25 | -0.16 |
| 11 | -0.05 | -0.08 | -0.01 | -0.17 | -0.22 | -0.12 |
| 12 | -0.05 | -0.09 | -0.01 | -0.22 | -0.28 | -0.17 |
| 13 | -0.04 | -0.08 | 0.00 | -0.20 | -0.26 | -0.14 |
| 14 | -0.02 | -0.07 | 0.02 | -0.18 | -0.25 | -0.11 |
| 15 | -0.08 | -0.13 | -0.03 | -0.22 | -0.29 | -0.14 |
| **SBP change since delivery** | | | | | | |
|  | Non GDM | | | GDM | | |
| Years since delivery | SBP change (mmHg) | 95% CI lower bound | 95% CI upper bound | SBP change (mmHg) | 95% CI lower bound | 95% CI upper bound |
| 1 | 0.70 | 0.59 | 0.81 | 1.14 | 0.93 | 1.34 |
| 2 | 1.26 | 1.14 | 1.37 | 1.81 | 1.59 | 2.03 |
| 3 | 1.97 | 1.84 | 2.09 | 2.55 | 2.31 | 2.78 |
| 4 | 2.64 | 2.50 | 2.78 | 3.38 | 3.12 | 3.63 |
| 5 | 3.29 | 3.13 | 3.44 | 4.03 | 3.75 | 4.32 |
| 6 | 3.86 | 3.69 | 4.02 | 4.59 | 4.28 | 4.90 |
| 7 | 4.47 | 4.28 | 4.65 | 5.18 | 4.84 | 5.52 |
| 8 | 5.21 | 5.00 | 5.41 | 6.02 | 5.64 | 6.39 |
| 9 | 5.70 | 5.47 | 5.92 | 6.50 | 6.09 | 6.92 |
| 10 | 6.60 | 6.36 | 6.85 | 7.25 | 6.79 | 7.71 |
| 11 | 6.73 | 6.45 | 7.00 | 7.45 | 6.92 | 7.98 |
| 12 | 7.35 | 7.05 | 7.66 | 8.09 | 7.49 | 8.69 |
| 13 | 7.76 | 7.41 | 8.10 | 8.62 | 7.94 | 9.29 |
| 14 | 8.70 | 8.31 | 9.08 | 9.01 | 8.23 | 9.80 |
| 15 | 9.35 | 8.91 | 9.78 | 8.10 | 7.22 | 8.99 |
| **DBP change since delivery** | | | | | | |
|  | Non GDM | | | GDM | | |
| Years since delivery | DBP change (mmHg) | 95% CI lower bound | 95% CI upper bound | DBP change (mmHg) | 95% CI lower bound | 95% CI upper bound |
| 1 | 0.05 | -0.03 | 0.13 | 0.39 | 0.24 | 0.54 |
| 2 | 0.51 | 0.42 | 0.59 | 0.97 | 0.81 | 1.13 |
| 3 | 1.15 | 1.06 | 1.25 | 1.69 | 1.51 | 1.86 |
| 4 | 1.72 | 1.62 | 1.83 | 2.27 | 2.07 | 2.46 |
| 5 | 2.14 | 2.03 | 2.26 | 2.74 | 2.53 | 2.95 |
| 6 | 2.60 | 2.47 | 2.72 | 3.05 | 2.82 | 3.28 |
| 7 | 2.99 | 2.85 | 3.13 | 3.43 | 3.17 | 3.68 |
| 8 | 3.49 | 3.34 | 3.64 | 3.77 | 3.49 | 4.05 |
| 9 | 3.62 | 3.45 | 3.78 | 3.96 | 3.66 | 4.27 |
| 10 | 4.14 | 3.96 | 4.32 | 4.51 | 4.17 | 4.85 |
| 11 | 4.18 | 3.98 | 4.38 | 4.86 | 4.47 | 5.26 |
| 12 | 4.67 | 4.44 | 4.90 | 4.45 | 4.01 | 4.90 |
| 13 | 4.87 | 4.61 | 5.13 | 4.77 | 4.27 | 5.27 |
| 14 | 4.97 | 4.68 | 5.26 | 4.92 | 4.34 | 5.50 |
| 15 | 5.76 | 5.44 | 6.09 | 4.53 | 3.88 | 5.19 |

## Supplementary Table 6: change from baseline at each follow-up year for women based on both GDM and T2DM status.

| **Weight change since delivery** | | | | | | | | | | | | |
| --- | --- | --- | --- | --- | --- | --- | --- | --- | --- | --- | --- | --- |
|  | **Non GDM - Non T2DM** | | | **Non GDM - T2DM** | | | **GDM - Non T2DM** | | | **GDM - T2DM** | | |
| Years since delivery | Weight change (Kg) | 95% CI lower bound | 95% CI upper bound | Weight change (Kg) | 95% CI lower bound | 95% CI upper bound | Weight change (Kg) | 95% CI lower bound | 95% CI upper bound | Weight change (Kg) | 95% CI lower bound | 95% CI upper bound |
| 1 | -1.16 | -1.26 | -1.06 | 2.42 | 1.52 | 3.31 | 0.09 | -0.10 | 0.28 | 1.22 | 0.84 | 1.61 |
| 2 | -0.35 | -0.45 | -0.24 | 4.16 | 3.27 | 5.05 | 0.63 | 0.43 | 0.83 | 1.91 | 1.52 | 2.30 |
| 3 | 0.28 | 0.17 | 0.40 | 4.32 | 3.41 | 5.22 | 1.17 | 0.95 | 1.38 | 2.04 | 1.64 | 2.44 |
| 4 | 0.65 | 0.53 | 0.77 | 5.68 | 4.77 | 6.59 | 1.34 | 1.10 | 1.58 | 2.08 | 1.66 | 2.49 |
| 5 | 1.17 | 1.04 | 1.30 | 5.78 | 4.86 | 6.70 | 1.83 | 1.57 | 2.09 | 1.97 | 1.53 | 2.41 |
| 6 | 1.65 | 1.51 | 1.79 | 7.02 | 6.07 | 7.96 | 2.44 | 2.16 | 2.72 | 2.13 | 1.67 | 2.58 |
| 7 | 2.12 | 1.96 | 2.27 | 7.14 | 6.16 | 8.12 | 2.85 | 2.54 | 3.16 | 2.28 | 1.80 | 2.76 |
| 8 | 2.56 | 2.39 | 2.73 | 7.44 | 6.41 | 8.48 | 3.10 | 2.76 | 3.44 | 2.32 | 1.81 | 2.83 |
| 9 | 3.03 | 2.85 | 3.22 | 7.39 | 6.34 | 8.45 | 3.35 | 2.97 | 3.73 | 2.50 | 1.96 | 3.04 |
| 10 | 3.53 | 3.33 | 3.73 | 8.67 | 7.55 | 9.79 | 3.70 | 3.29 | 4.12 | 2.35 | 1.76 | 2.94 |
| 11 | 4.14 | 3.92 | 4.37 | 8.16 | 6.94 | 9.38 | 3.79 | 3.29 | 4.29 | 2.72 | 2.08 | 3.37 |
| 12 | 4.56 | 4.32 | 4.81 | 10.42 | 9.18 | 11.66 | 4.54 | 3.98 | 5.10 | 2.69 | 1.98 | 3.41 |
| 13 | 4.90 | 4.62 | 5.18 | 10.46 | 9.02 | 11.90 | 4.33 | 3.68 | 4.97 | 3.28 | 2.47 | 4.09 |
| 14 | 5.49 | 5.18 | 5.80 | 11.38 | 9.78 | 12.97 | 5.06 | 4.35 | 5.77 | 3.59 | 2.65 | 4.53 |
| 15 | 5.94 | 5.58 | 6.30 | 10.82 | 8.94 | 12.70 | 5.21 | 4.37 | 6.04 | 3.65 | 2.62 | 4.69 |
| **HbA1c change since delivery** | | | | | | | | | | | | |
|  | **Non GDM - Non T2DM** | | | **Non GDM - T2DM** | | | **GDM - Non T2DM** | | | **GDM - T2DM** | | |
| Years since delivery | HbA1c change (mmol/mol) | 95% CI lower bound | 95% CI upper bound | HbA1c change (mmol/mol) | 95% CI lower bound | 95% CI upper bound | HbA1c change (mmol/mol) | 95% CI lower bound | 95% CI upper bound | HbA1c change (mmol/mol) | 95% CI lower bound | 95% CI upper bound |
| 1 | 0.15 | 0.03 | 0.27 | 0.75 | -0.52 | 2.03 | 0.35 | 0.22 | 0.47 | 4.19 | 3.84 | 4.53 |
| 2 | 0.15 | 0.03 | 0.28 | 3.22 | 2.02 | 4.41 | 0.43 | 0.30 | 0.57 | 6.13 | 5.78 | 6.47 |
| 3 | 0.13 | 0.00 | 0.26 | 3.89 | 2.70 | 5.08 | 0.58 | 0.43 | 0.73 | 7.57 | 7.22 | 7.92 |
| 4 | 0.13 | -0.01 | 0.26 | 5.48 | 4.28 | 6.68 | 0.70 | 0.54 | 0.86 | 8.69 | 8.33 | 9.05 |
| 5 | 0.20 | 0.05 | 0.34 | 7.49 | 6.32 | 8.67 | 0.89 | 0.71 | 1.07 | 9.44 | 9.07 | 9.82 |
| 6 | 0.27 | 0.12 | 0.42 | 8.58 | 7.39 | 9.78 | 0.94 | 0.74 | 1.13 | 10.76 | 10.38 | 11.15 |
| 7 | 0.39 | 0.23 | 0.55 | 8.32 | 7.11 | 9.52 | 1.19 | 0.98 | 1.41 | 11.89 | 11.49 | 12.28 |
| 8 | 0.53 | 0.36 | 0.70 | 9.82 | 8.58 | 11.05 | 1.30 | 1.07 | 1.54 | 13.00 | 12.59 | 13.41 |
| 9 | 0.67 | 0.49 | 0.85 | 10.59 | 9.36 | 11.82 | 1.57 | 1.30 | 1.83 | 14.36 | 13.93 | 14.79 |
| 10 | 0.82 | 0.63 | 1.01 | 12.42 | 11.18 | 13.66 | 1.75 | 1.46 | 2.04 | 14.77 | 14.31 | 15.22 |
| 11 | 1.07 | 0.86 | 1.28 | 12.30 | 11.03 | 13.57 | 1.78 | 1.45 | 2.11 | 15.86 | 15.37 | 16.35 |
| 12 | 1.26 | 1.03 | 1.49 | 13.18 | 11.88 | 14.47 | 1.98 | 1.61 | 2.36 | 16.42 | 15.88 | 16.96 |
| 13 | 1.41 | 1.16 | 1.66 | 14.81 | 13.43 | 16.18 | 2.17 | 1.74 | 2.60 | 17.68 | 17.08 | 18.28 |
| 14 | 1.69 | 1.42 | 1.96 | 16.36 | 14.94 | 17.79 | 2.69 | 2.21 | 3.17 | 19.75 | 19.09 | 20.41 |
| 15 | 1.80 | 1.50 | 2.10 | 14.37 | 12.81 | 15.94 | 2.84 | 2.30 | 3.39 | 19.66 | 18.89 | 20.42 |
| **LDL cholesterol change since delivery** | | | | | | | | | | | | |
|  | **Non GDM - Non T2DM** | | | **Non GDM - T2DM** | | | **GDM - Non T2DM** | | | **GDM - T2DM** | | |
| Years since delivery | LDL cholesterol (mmol/l) | 95% CI lower bound | 95% CI upper bound | LDL cholesterol (mmol/l) | 95% CI lower bound | 95% CI upper bound | LDL cholesterol (mmol/l) | 95% CI lower bound | 95% CI upper bound | LDL cholesterol (mmol/l) | 95% CI lower bound | 95% CI upper bound |
| 1 | -0.21 | -0.24 | -0.19 | -0.20 | -0.36 | -0.04 | -0.18 | -0.21 | -0.15 | -0.16 | -0.21 | -0.10 |
| 2 | -0.18 | -0.21 | -0.16 | -0.10 | -0.26 | 0.05 | -0.15 | -0.18 | -0.11 | -0.13 | -0.18 | -0.07 |
| 3 | -0.15 | -0.17 | -0.12 | -0.15 | -0.31 | 0.00 | -0.12 | -0.16 | -0.09 | -0.20 | -0.26 | -0.15 |
| 4 | -0.15 | -0.18 | -0.12 | -0.17 | -0.32 | -0.02 | -0.13 | -0.17 | -0.10 | -0.21 | -0.27 | -0.15 |
| 5 | -0.14 | -0.17 | -0.12 | -0.14 | -0.29 | 0.01 | -0.13 | -0.17 | -0.09 | -0.23 | -0.29 | -0.17 |
| 6 | -0.11 | -0.14 | -0.08 | -0.32 | -0.47 | -0.16 | -0.09 | -0.13 | -0.05 | -0.28 | -0.34 | -0.22 |
| 7 | -0.10 | -0.13 | -0.07 | -0.27 | -0.43 | -0.12 | -0.08 | -0.13 | -0.04 | -0.29 | -0.35 | -0.23 |
| 8 | -0.09 | -0.12 | -0.06 | -0.23 | -0.39 | -0.07 | -0.08 | -0.12 | -0.03 | -0.34 | -0.40 | -0.27 |
| 9 | -0.06 | -0.09 | -0.03 | -0.34 | -0.49 | -0.18 | -0.04 | -0.10 | 0.01 | -0.39 | -0.46 | -0.32 |
| 10 | -0.04 | -0.08 | -0.01 | -0.40 | -0.56 | -0.24 | -0.06 | -0.12 | -0.01 | -0.40 | -0.47 | -0.33 |
| 11 | -0.01 | -0.05 | 0.02 | -0.28 | -0.45 | -0.12 | -0.13 | -0.19 | -0.06 | -0.35 | -0.43 | -0.27 |
| 12 | 0.04 | 0.00 | 0.08 | -0.39 | -0.57 | -0.22 | -0.08 | -0.15 | 0.00 | -0.41 | -0.50 | -0.33 |
| 13 | 0.04 | -0.01 | 0.08 | -0.40 | -0.60 | -0.21 | 0.02 | -0.06 | 0.11 | -0.45 | -0.55 | -0.36 |
| 14 | 0.01 | -0.04 | 0.06 | -0.33 | -0.55 | -0.11 | -0.03 | -0.12 | 0.07 | -0.53 | -0.63 | -0.42 |
| 15 | -0.01 | -0.07 | 0.04 | -0.58 | -0.84 | -0.32 | 0.00 | -0.10 | 0.09 | -0.67 | -0.79 | -0.55 |
| **HDL cholesterol change since delivery** | | | | | | | | | | | | |
|  | **Non GDM - Non T2DM** | | | **Non GDM - T2DM** | | | **GDM - Non T2DM** | | | **GDM - T2DM** | | |
| Years since delivery | HDL cholesterol (mmol/l) | 95% CI lower bound | 95% CI upper bound | HDL cholesterol (mmol/l) | 95% CI lower bound | 95% CI upper bound | HDL cholesterol (mmol/l) | 95% CI lower bound | 95% CI upper bound | HDL cholesterol (mmol/l) | 95% CI lower bound | 95% CI upper bound |
| 1 | -0.04 | -0.04 | -0.03 | -0.03 | -0.09 | 0.02 | -0.05 | -0.06 | -0.04 | -0.03 | -0.05 | -0.02 |
| 2 | -0.02 | -0.03 | -0.01 | -0.04 | -0.09 | 0.01 | -0.04 | -0.06 | -0.03 | -0.03 | -0.05 | -0.01 |
| 3 | 0.00 | -0.01 | 0.00 | -0.06 | -0.12 | -0.01 | -0.04 | -0.05 | -0.02 | -0.03 | -0.04 | -0.01 |
| 4 | 0.02 | 0.01 | 0.02 | -0.05 | -0.10 | 0.00 | -0.02 | -0.03 | -0.01 | -0.02 | -0.04 | 0.00 |
| 5 | 0.02 | 0.01 | 0.03 | -0.03 | -0.08 | 0.02 | -0.01 | -0.02 | 0.00 | 0.00 | -0.02 | 0.01 |
| 6 | 0.04 | 0.03 | 0.05 | -0.03 | -0.08 | 0.02 | 0.00 | -0.01 | 0.01 | -0.02 | -0.04 | 0.00 |
| 7 | 0.05 | 0.04 | 0.06 | -0.02 | -0.07 | 0.03 | -0.01 | -0.02 | 0.01 | 0.01 | -0.01 | 0.03 |
| 8 | 0.05 | 0.04 | 0.06 | -0.01 | -0.06 | 0.04 | 0.02 | 0.00 | 0.03 | 0.01 | -0.02 | 0.03 |
| 9 | 0.08 | 0.07 | 0.09 | -0.01 | -0.06 | 0.04 | 0.02 | 0.00 | 0.04 | 0.01 | -0.01 | 0.03 |
| 10 | 0.07 | 0.06 | 0.08 | 0.01 | -0.04 | 0.07 | 0.04 | 0.03 | 0.06 | 0.02 | 0.00 | 0.05 |
| 11 | 0.07 | 0.06 | 0.08 | -0.01 | -0.06 | 0.05 | 0.04 | 0.02 | 0.06 | 0.02 | -0.01 | 0.04 |
| 12 | 0.07 | 0.06 | 0.09 | 0.02 | -0.04 | 0.08 | 0.06 | 0.04 | 0.09 | 0.03 | 0.00 | 0.06 |
| 13 | 0.08 | 0.07 | 0.09 | 0.01 | -0.05 | 0.07 | 0.08 | 0.05 | 0.11 | 0.02 | -0.01 | 0.05 |
| 14 | 0.08 | 0.06 | 0.09 | 0.01 | -0.06 | 0.07 | 0.05 | 0.02 | 0.08 | 0.02 | -0.01 | 0.05 |
| 15 | 0.10 | 0.08 | 0.11 | -0.01 | -0.08 | 0.06 | 0.05 | 0.02 | 0.08 | 0.02 | -0.02 | 0.05 |
| **Total cholesterol change since delivery** | | | | | | | | | | | | |
|  | **Non GDM - Non T2DM** | | | **Non GDM - T2DM** | | | **GDM - Non T2DM** | | | **GDM - T2DM** | | |
| Years since delivery | Total cholesterol (mmol/l) | 95% CI lower bound | 95% CI upper bound | Total cholesterol (mmol/l) | 95% CI lower bound | 95% CI upper bound | Total cholesterol (mmol/l) | 95% CI lower bound | 95% CI upper bound | Total cholesterol (mmol/l) | 95% CI lower bound | 95% CI upper bound |
| 1 | -0.28 | -0.31 | -0.26 | -0.27 | -0.30 | -0.24 | -0.27 | -0.30 | -0.24 | -0.22 | -0.28 | -0.17 |
| 2 | -0.24 | -0.26 | -0.21 | -0.24 | -0.27 | -0.20 | -0.24 | -0.27 | -0.20 | -0.21 | -0.26 | -0.16 |
| 3 | -0.18 | -0.20 | -0.16 | -0.20 | -0.24 | -0.17 | -0.20 | -0.24 | -0.17 | -0.24 | -0.29 | -0.19 |
| 4 | -0.15 | -0.18 | -0.13 | -0.18 | -0.21 | -0.15 | -0.18 | -0.21 | -0.15 | -0.27 | -0.33 | -0.22 |
| 5 | -0.12 | -0.14 | -0.09 | -0.16 | -0.20 | -0.13 | -0.16 | -0.20 | -0.13 | -0.27 | -0.32 | -0.21 |
| 6 | -0.08 | -0.10 | -0.05 | -0.10 | -0.14 | -0.07 | -0.10 | -0.14 | -0.07 | -0.27 | -0.33 | -0.21 |
| 7 | -0.03 | -0.06 | 0.00 | -0.07 | -0.12 | -0.03 | -0.07 | -0.12 | -0.03 | -0.29 | -0.35 | -0.23 |
| 8 | -0.01 | -0.03 | 0.02 | -0.05 | -0.10 | -0.01 | -0.05 | -0.10 | -0.01 | -0.29 | -0.35 | -0.23 |
| 9 | 0.04 | 0.01 | 0.07 | 0.00 | -0.05 | 0.05 | 0.00 | -0.05 | 0.05 | -0.33 | -0.40 | -0.27 |
| 10 | 0.08 | 0.05 | 0.11 | 0.00 | -0.05 | 0.05 | 0.00 | -0.05 | 0.05 | -0.35 | -0.41 | -0.28 |
| 11 | 0.13 | 0.10 | 0.17 | -0.01 | -0.07 | 0.05 | -0.01 | -0.07 | 0.05 | -0.30 | -0.38 | -0.23 |
| 12 | 0.18 | 0.15 | 0.22 | 0.09 | 0.03 | 0.15 | 0.09 | 0.03 | 0.15 | -0.37 | -0.45 | -0.29 |
| 13 | 0.23 | 0.19 | 0.27 | 0.14 | 0.06 | 0.21 | 0.14 | 0.06 | 0.21 | -0.38 | -0.46 | -0.29 |
| 14 | 0.23 | 0.19 | 0.27 | 0.12 | 0.04 | 0.20 | 0.12 | 0.04 | 0.20 | -0.38 | -0.48 | -0.28 |
| 15 | 0.23 | 0.19 | 0.28 | 0.18 | 0.09 | 0.27 | 0.18 | 0.09 | 0.27 | -0.59 | -0.71 | -0.48 |
| **Total : HDL cholesterol ratio change since delivery** | | | | | | | | | | | | |
|  | **Non GDM - Non T2DM** | | | **Non GDM - T2DM** | | | **GDM - Non T2DM** | | | **GDM - T2DM** | | |
| Years since delivery | Ratio change (mmol/l) | 95% CI lower bound | 95% CI upper bound | Ratio change (mmol/l) | 95% CI lower bound | 95% CI upper bound | Ratio change (mmol/l) | 95% CI lower bound | 95% CI upper bound | Ratio change (mmol/l) | 95% CI lower bound | 95% CI upper bound |
| 1 | -0.13 | -0.16 | -0.10 | -0.07 | -0.24 | 0.09 | -0.10 | -0.13 | -0.06 | -0.06 | -0.11 | 0.00 |
| 2 | -0.12 | -0.15 | -0.10 | 0.06 | -0.09 | 0.22 | -0.07 | -0.10 | -0.04 | -0.05 | -0.10 | 0.01 |
| 3 | -0.12 | -0.14 | -0.09 | 0.18 | 0.02 | 0.34 | -0.06 | -0.10 | -0.02 | -0.12 | -0.17 | -0.06 |
| 4 | -0.14 | -0.16 | -0.11 | 0.13 | -0.02 | 0.29 | -0.09 | -0.13 | -0.05 | -0.15 | -0.21 | -0.10 |
| 5 | -0.13 | -0.16 | -0.11 | 0.04 | -0.11 | 0.19 | -0.12 | -0.16 | -0.08 | -0.20 | -0.26 | -0.14 |
| 6 | -0.13 | -0.16 | -0.11 | -0.01 | -0.17 | 0.15 | -0.08 | -0.12 | -0.04 | -0.17 | -0.23 | -0.10 |
| 7 | -0.12 | -0.15 | -0.09 | 0.04 | -0.12 | 0.20 | -0.06 | -0.10 | -0.01 | -0.26 | -0.33 | -0.20 |
| 8 | -0.10 | -0.13 | -0.07 | 0.03 | -0.14 | 0.19 | -0.10 | -0.15 | -0.05 | -0.26 | -0.33 | -0.20 |
| 9 | -0.14 | -0.17 | -0.11 | -0.11 | -0.27 | 0.05 | -0.06 | -0.12 | -0.01 | -0.28 | -0.35 | -0.21 |
| 10 | -0.09 | -0.12 | -0.06 | -0.25 | -0.41 | -0.08 | -0.14 | -0.19 | -0.08 | -0.38 | -0.46 | -0.31 |
| 11 | -0.04 | -0.08 | -0.01 | -0.13 | -0.30 | 0.04 | -0.14 | -0.20 | -0.08 | -0.30 | -0.38 | -0.22 |
| 12 | -0.03 | -0.07 | 0.01 | -0.31 | -0.48 | -0.13 | -0.14 | -0.21 | -0.07 | -0.43 | -0.51 | -0.34 |
| 13 | -0.03 | -0.07 | 0.02 | -0.26 | -0.45 | -0.07 | -0.08 | -0.16 | 0.00 | -0.43 | -0.52 | -0.33 |
| 14 | -0.02 | -0.06 | 0.03 | -0.18 | -0.38 | 0.02 | -0.09 | -0.17 | 0.00 | -0.40 | -0.50 | -0.30 |
| 15 | -0.07 | -0.12 | -0.02 | -0.32 | -0.55 | -0.09 | -0.02 | -0.12 | 0.07 | -0.60 | -0.72 | -0.48 |
| **SBP change since delivery** | | | | | | | | | | | | |
|  | **Non GDM - Non T2DM** | | | **Non GDM - T2DM** | | | **GDM - Non T2DM** | | | **GDM - T2DM** | | |
| Years since delivery | SBP change (mmHg) | 95% CI lower bound | 95% CI upper bound | SBP change (mmHg) | 95% CI lower bound | 95% CI upper bound | SBP change (mmHg) | 95% CI lower bound | 95% CI upper bound | SBP change (mmHg) | 95% CI lower bound | 95% CI upper bound |
| 1 | 0.69 | 0.58 | 0.79 | 1.50 | 0.30 | 2.69 | 1.00 | 0.78 | 1.22 | 1.24 | 0.72 | 1.77 |
| 2 | 1.24 | 1.12 | 1.36 | 1.80 | 0.58 | 3.01 | 1.67 | 1.43 | 1.90 | 1.78 | 1.25 | 2.32 |
| 3 | 1.94 | 1.81 | 2.06 | 3.18 | 1.96 | 4.41 | 2.36 | 2.10 | 2.62 | 2.55 | 1.99 | 3.10 |
| 4 | 2.61 | 2.47 | 2.75 | 3.61 | 2.36 | 4.86 | 3.35 | 3.06 | 3.64 | 2.72 | 2.14 | 3.31 |
| 5 | 3.27 | 3.11 | 3.42 | 3.47 | 2.21 | 4.74 | 4.02 | 3.70 | 4.34 | 3.32 | 2.71 | 3.92 |
| 6 | 3.82 | 3.65 | 3.99 | 4.52 | 3.21 | 5.84 | 4.58 | 4.23 | 4.94 | 3.82 | 3.18 | 4.46 |
| 7 | 4.45 | 4.26 | 4.63 | 4.56 | 3.19 | 5.92 | 5.20 | 4.80 | 5.59 | 4.32 | 3.64 | 5.00 |
| 8 | 5.18 | 4.97 | 5.39 | 5.41 | 4.01 | 6.82 | 5.95 | 5.51 | 6.40 | 5.34 | 4.61 | 6.07 |
| 9 | 5.72 | 5.49 | 5.95 | 4.10 | 2.66 | 5.55 | 6.70 | 6.20 | 7.20 | 5.27 | 4.50 | 6.05 |
| 10 | 6.61 | 6.36 | 6.86 | 5.63 | 4.11 | 7.15 | 7.39 | 6.82 | 7.95 | 6.15 | 5.31 | 6.99 |
| 11 | 6.68 | 6.40 | 6.96 | 7.35 | 5.70 | 9.00 | 7.15 | 6.50 | 7.80 | 7.16 | 6.21 | 8.10 |
| 12 | 7.29 | 6.98 | 7.61 | 8.33 | 6.57 | 10.09 | 8.47 | 7.73 | 9.20 | 6.54 | 5.49 | 7.59 |
| 13 | 7.69 | 7.34 | 8.05 | 8.88 | 6.94 | 10.83 | 9.12 | 8.29 | 9.94 | 6.81 | 5.63 | 7.98 |
| 14 | 8.70 | 8.30 | 9.09 | 8.19 | 5.99 | 10.39 | 9.89 | 8.93 | 10.85 | 6.48 | 5.11 | 7.84 |
| 15 | 9.26 | 8.82 | 9.71 | 11.41 | 8.87 | 13.94 | 8.67 | 7.59 | 9.75 | 6.11 | 4.56 | 7.67 |
| **DBP change since delivery** | | | | | | | | | | | | |
|  | **Non GDM - Non T2DM** | | | **Non GDM - T2DM** | | | **GDM - Non T2DM** | | | **GDM - T2DM** | | |
| Years since delivery | DBP change (mmHg) | 95% CI lower bound | 95% CI upper bound | DBP change (mmHg) | 95% CI lower bound | 95% CI upper bound | DBP change (mmHg) | 95% CI lower bound | 95% CI upper bound | DBP change (mmHg) | 95% CI lower bound | 95% CI upper bound |
| 1 | 0.03 | -0.05 | 0.11 | 1.48 | 0.60 | 2.37 | 0.29 | 0.13 | 0.45 | 0.56 | 0.18 | 0.95 |
| 2 | 0.49 | 0.40 | 0.57 | 1.79 | 0.89 | 2.70 | 0.85 | 0.68 | 1.03 | 1.11 | 0.71 | 1.51 |
| 3 | 1.14 | 1.04 | 1.23 | 1.84 | 0.92 | 2.75 | 1.63 | 1.44 | 1.83 | 1.51 | 1.10 | 1.92 |
| 4 | 1.71 | 1.60 | 1.81 | 2.28 | 1.36 | 3.21 | 2.24 | 2.02 | 2.45 | 1.95 | 1.52 | 2.38 |
| 5 | 2.11 | 2.00 | 2.23 | 3.44 | 2.50 | 4.38 | 2.88 | 2.64 | 3.12 | 1.87 | 1.42 | 2.32 |
| 6 | 2.58 | 2.45 | 2.71 | 3.28 | 2.31 | 4.25 | 3.17 | 2.90 | 3.43 | 2.28 | 1.80 | 2.76 |
| 7 | 2.97 | 2.83 | 3.11 | 3.60 | 2.58 | 4.61 | 3.63 | 3.33 | 3.93 | 2.43 | 1.93 | 2.94 |
| 8 | 3.47 | 3.32 | 3.62 | 4.03 | 2.99 | 5.08 | 3.95 | 3.62 | 4.28 | 2.84 | 2.29 | 3.38 |
| 9 | 3.62 | 3.45 | 3.79 | 3.33 | 2.26 | 4.41 | 4.17 | 3.80 | 4.54 | 2.99 | 2.42 | 3.57 |
| 10 | 4.16 | 3.97 | 4.34 | 3.56 | 2.43 | 4.69 | 4.96 | 4.54 | 5.37 | 3.09 | 2.47 | 3.71 |
| 11 | 4.16 | 3.95 | 4.37 | 4.69 | 3.46 | 5.91 | 5.20 | 4.71 | 5.68 | 3.64 | 2.94 | 4.35 |
| 12 | 4.64 | 4.41 | 4.87 | 5.33 | 4.02 | 6.64 | 5.15 | 4.60 | 5.69 | 2.57 | 1.79 | 3.35 |
| 13 | 4.88 | 4.61 | 5.14 | 4.63 | 3.18 | 6.08 | 5.52 | 4.90 | 6.13 | 2.78 | 1.91 | 3.66 |
| 14 | 4.97 | 4.68 | 5.27 | 4.86 | 3.22 | 6.49 | 5.71 | 4.99 | 6.42 | 2.82 | 1.80 | 3.84 |
| 15 | 5.69 | 5.36 | 6.02 | 7.73 | 5.85 | 9.62 | 5.10 | 4.30 | 5.91 | 2.83 | 1.67 | 3.98 |

## Supplementary Figure 3: Absolute and difference from first year postpartum for total cholesterol, HDL cholesterol, and total: HDL cholesterol ratio among women with or without GDM in pregnancy.

## Supplementary Figure 4: Absolute and difference from first year postpartum for total cholesterol, HDL cholesterol, and total: HDL cholesterol ratios among women with or without GDM in pregnancy who did or did not develop type 2 diabetes in pregnancy.
